# The Gut Commensal *Butyricimonas Virosa* Modulates Gut Microbiota‐Dependent Thiamine Metabolism and Attenuates Mouse Steatotic Liver Disease

**DOI:** 10.1002/advs.202517596

**Published:** 2026-01-20

**Authors:** Ningning He, Haoyu Wang, Zizhen Yang, Hui Li, Bei Liu, Kaiwei Chen, Zhinan Wu, Xinnan Zhao, Hewei Liang, Mengmeng Wang, Xiaofang Li, Yiyi Zhong, Haifeng Zhang, Liang Xiao, Karsten Kristiansen, Jixing Peng, Yuanqiang Zou, Shangyong Li

**Affiliations:** ^1^ School of Basic Medicine, Qingdao Medical College Qingdao University Qingdao China; ^2^ State Key Laboratory of Genome and Multi‐omics Technologies BGI Research Shenzhen China; ^3^ College of Life Sciences University of Chinese Academy of Sciences Beijing China; ^4^ Shenzhen Key Laboratory of Human commensal microorganisms and Health Research, BGI Research Shenzhen China; ^5^ BGI Precision Nutrition Shenzhen China; ^6^ Laboratory of Integrative Biomedicine, Department of Biology University of Copenhagen Copenhagen Denmark; ^7^ Shenzhen Key Laboratory of Human commensal microorganisms and Health Research Shenzhen China

**Keywords:** butyricimonas virosa, gut microbiota, MASLD, stachyose, thiamine metabolism

## Abstract

Metabolic dysfunction‐associated steatotic liver disease (MASLD) is a common chronic liver disease. This study investigates the anti‐MASLD effects of dietary prebiotic stachyose (STA) on disease progression identifying *Butyricimonas virosa* as a key bacterium boosted by STA supplementation. Oral gavage of *B. virosa* to high fat diet (HFD)‐fed mice significantly suppresses the progression of MASLD and modulates gut microbiota composition. Integration of metagenomic and metabolomic data demonstrates that *B. virosa* treatment significantly enhances the production of thiamine monophosphate (TMP), as well as its conversion to thiamine and subsequent accumulation in the liver. The accumulation of hepatic thiamine further leads to elevated thiamine pyrophosphate (TPP) concentrations enhancing the activity of branched‐chain α‐keto acid dehydrogenase E1 subunit α (BCKDHA) associated with augmented degradation of branched chain amino acids (BCAAs). Administration of *B. virosa* compensates via production of gut bacterial‐derived TMP for hepatic TPP deficiency in mice fed a thiamine‐deficient HFD. A population‐based analysis reveals an inverse correlation between plasma thiamine levels, abundances of bacterial genes involved in thiamine synthesis and metabolism, and phenotypes associated with MASLD, suggesting that key genes involved in fecal thiamine metabolism, as well as serum thiamine determination, may potentially serve as biomarkers for the diagnosis of MASLD.

## Graphical abstract

This study reveals that dietary stachyose enriches *Butyricimonas virosa*, effectively attenuating metabolic dysfunction‐associated steatotic liver disease (MASLD). Mechanistically, *B. virosa* enhances gut thiamine monophosphate synthesis, which elevates hepatic thiamine pyrophosphate levels. This metabolic restoration promotes branched‐chain amino acid degradation to mitigate hepatic steatosis and inflammation, highlighting a promising microbiota‐targeted therapeutic strategy.

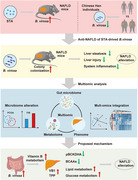

**Abbreviations**
MASLD: metabolic dysfunction‐associated steatotic liver disease
HFD: high‐fat diet
NCD: normal chow diet
STA: stachyose
*B. virosa*
: *Butyricimonas virosa*
TPP: Thiamine pyrophosphate
BCKDH: branched‐chain keto acid dehydrogenase
BCKDHA: branched‐chain α‐keto acid dehydrogenase E1 subunit α
GLP‐1: glucagon‐like peptide‐1
BCAAs: branched‐chain amino acids
AUC: area under the curve
IPGTT: intraperitoneal glucose tolerance test
HOMA‐IR: homeostatic model assessment for insulin resistance
eWAT: epididymal white adipose tissue
H&E: hematoxylin and eosin
TNF‐α: tumor necrosis factor‐alpha
IL‐1β: interleukin‐1β
IL‐6: interleukin‐6
IL‐10: interleukin‐10
ALT: alanine aminotransferase
AST: aspartate aminotransferase
TG: triglyceride
TC: total cholesterol
BMI: body mass index
HFTD: high‐fat thiamine‐deficient.

## 1 Introduction

Metabolic dysfunction‐associated steatotic liver disease (MASLD) represents the most prevalent hepatic disorder afflicting approximately 30% of the global population [1]. MASLD has the potential to progress to severe complications, including fibrosis, cirrhosis, and even hepatocellular carcinoma [2, 3]. The pathogenesis of MASLD is intricate, encompassing a spectrum of pathological processes such as lipid metabolism disorders, insulin resistance, oxidative stress, and dysbiosis of the gut microbiota [4]. Recent advances in metagenomics and metabolomics have significantly enhanced our understanding of how prebiotics and probiotics can modulate the gut microbiota and associated metabolites, contributing to the alleviation of MASLD [5].

The tetrasaccharide, stachyose (STA), has emerged as a prebiotic with the ability to modulate both the composition and functional capacity of the gut microbiota [6, 7, 8]. Our previous research has demonstrated that STA can mitigate symptoms associated with high‐fat diet (HFD)‐induced obesity in mice, potentially linked to alterations in gut microbiota and their metabolic products [9]. However, the specific bacterial populations enriched and the metabolic pathways that underpin potential anti‐MASLD effects of STA have yet to be fully elucidated.

Among gut bacterial species associated with health beneficial effects, *Akkermansia muciniphila* and *Faecalibacterium prausnitzii* stand out [10, 11, 12]*. Butyricimonas virosa* (*B. virosa*) was originally isolated from rat feces and previously shown to produce butyrate and isobutyrate in vitro [13]. Subsequent investigations have indicated that *B. virosa* holds promises as a potential probiotic affecting glucose regulation by activating the hepatic glucagon‐like peptide‐1 (GLP‐1) receptor rather than primarily through gut colonization or butyrate production [14]. Still, the precise mechanisms by which *B. virosa* impinges on metabolism, and especially MASLD remain poorly understood.

The gut microbiota plays a vital role in shaping host physiology through the production of metabolites and also functions as an alternative source of B vitamins (Vit B), distinct from dietary intake [15]. Patients with MASLD frequently exhibit dysbiosis of the gut microbiota, which may lead to a diminished capacity for Vit B synthesis [16, 17, 18]. Thiamine (Vit B1) is key in maintaining cellular health and energy metabolism. A sustained lack of dietary intake, especially in high‐risk groups such as those engaged in long‐haul voyages or prolonged offshore work where fresh food access is restricted, can rapidly lead to severe neurological and cardiovascular disorders [19, 20]. In contrast to dietary thiamine, which is absorbed directly in the small intestine, the gut microbiota primarily generates thiamine monophosphate (TMP) [21]. TMP can be converted into thiamine pyrophosphate (TPP) by the gut microbiota, allowing for absorption and utilization by the intestinal epithelium. Alternatively, TMP can be transformed into thiamine, which then enters the liver through the enterohepatic circulation to exert its physiological effects [22, 23]. Recent studies have demonstrated that oral thiamine supplementation can alleviate symptoms of MASLD [24]. However, the beneficial effects of gut microbiota‐derived TMP and thiamine on liver function and the underlying mechanisms remain to be elucidated.

In this study, we demonstrate that dietary intake of STA is associated with an enrichment of *B. virosa* linked to an inhibition of the progression of HFD‐induced MASLD in mice. Our findings point to a potential mechanism by which *B. virosa* modulates the microbiota to synthesize TMP, thereby enhancing hepatic TPP concentration and promoting the degradation of branched‐chain amino acids (BCAAs). These findings indicate that supplementation with *B. virosa* and modulation of gut microbiota‐derived thiamine may serve as a potential adjuvant therapy for managing MASLD.

## 2 Material and Methods

### 2.1 Materials

STA (SS9461) with purity ≥98% was acquired from Solarbio Life Sciences (Beijing, China). *B. virosa* AM16‐14 was isolated and cultured by BGI‐Shenzhen (Shenzhen, China). The antibodies against BCKDHA (A21588) and β‐Actin (AC026) were obtained from ABclonal Biotechnology Co., Ltd. (Wuhan, China). The antibody against Phospho‐BCKDHA (AF3826) was purchased from Affinity Biosciences (Jiangsu, China). Bacimethrin (BD587635) was purchased from Bide (Shanghai, China). Ampicillin (A910962), metronidazole (M813526), vancomycin (V871983), neomycin (N799581) and TMP (B781585) were obtained from Macklin Inc. (Shanghai, China). Additional materials are listed in the Supplemental Materials and Methods.

### 2.2 Animals and Treatment

All experiments on animals were performed on the basis of the Guide for the Care and Use of Laboratory Animals of Qingdao University, and the experiments were approved by the Animal Ethics Committee of the Medical College of Qingdao University (QDU‐AEC‐2022361, QDU‐AEC‐2023389 and QDU‐AEC‐2024777). The mice were housed in standard cages at a temperature range of 22–24°C, under a 12 h light‐dark cycle. After one week of acclimatization, mice were randomly divided into experimental groups (Supporting Information and Methods). Bacterial suspensions were prepared by inoculating *B. virosa* into MPYG broth and culturing to the mid‐logarithmic phase. Viable bacterial counts were counted using a hemocytometer. *B. virosa* was suspended in PBS to a concentration of 1 × 10^10^ CFU/mL. Mice were gavaged daily with 100 µL of the suspension, corresponding to a daily dose of 1 × 10^9^ CFU/mouse as previously described [25]. All suspensions were freshly prepared before gavage and gently vortexed to ensure uniform distribution. Throughout the experimental period, daily monitoring, including measurements of body weight, was performed. Before the end of the experiment, fecal samples were collected. After an 8 h fasting period, blood samples were obtained under isoflurane anesthesia. The blood was centrifuged to collect serum samples. Following dissection, liver tissue and epididymal white adipose tissues (eWAT) were collected, weighed, and subsequently frozen at −80°C for future analysis.

### 2.3 In Vitro Bacteria Culture

The *B. virosa* has been deposited at BGI Research with the accession number AM16‐14 [26, 27]. The strain was authenticated via 16S rRNA gene sequencing, confirming its taxonomic identity and purity. *B. virosa* AM16‐14 were cultured in MPYG broth as previously described [28]. The addition of STA to 1% w/v was selected based on preliminary experiments and utilized to assess the impact on the proliferation of *B. virosa*. The STA degradation products were subsequently analyzed using an ultrahigh performance liquid chromatography‐tandem mass spectrometry (UPLC‐MS/MS) system (ACQUITY UPLC‐Xevo TQ‐S, Waters, USA) with an Ilite Supersil NH_2_‐S amino‐bonded column (4.6 mm × 250 mm, 5 µm) [29, 30].

### 2.4 Glucose Metabolism and Serum Insulin Detection

Following fasting overnight, an intraperitoneal glucose tolerance test (IPGTT) was performed. Blood glucose concentrations were measured using a glucometer, and blood samples were taken from tip of the tail at 0, 30, 60, 90 and 120 min after intraperitoneally injection of glucose (2 g/kg body weight). The area under the curve (AUC) was calculated as described previously [31]. Fasting insulin levels in serum were detected using an Elisa kit (Beyotime Biotechnology, Shanghai, China).

### 2.5 Histological Analysis

Liver and adipose tissues were fixed in 10% formalin at room temperature for 24 h, followed by embedding in paraffin and sectioning into 5 µm thick sections. Liver tissues were stained with hematoxylin and eosin (H&E) or oil red O; eWAT tissues were stained with H&E. The image analysis software ImageJ (v1.53a, MD, USA) was used to analyze liver and adipose tissue sections. The severity of liver lesions was assessed using the MASLD activity score (0–8) comprising liver steatosis (scale of 0–3), lobular inflammation (scale of 0–3), and hepatocellular ballooning (scale of 0–2) [32].

### 2.6 Inflammatory Cytokines Analysis

The concentrations of pro‐inflammatory cytokines (TNF‐α, IL‐1β, and IL‐6) and the anti‐inflammatory cytokine (IL‐10) in serum were measured by using an ELISA kit (Thermo Fisher Scientific, Inc., Waltham, MA, USA).

### 2.7 Serum Biochemical Analysis and Liver Function Tests

Analyses of serum triglyceride (TG), total cholesterol (TC), alanine aminotransferase (ALT), aspartate aminotransferase (AST) were performed using a fully automated biochemical analyzer (Chemray 800, Rayto Life and Analytical Sciences Co., Ltd, Shenzhen, China). Nine volumes of saline were added to liver samples which were subsequently homogenized at 70 Hz in a High‐speed Low‐temperature Tissue Homogenizer (KZ‐III‐F, Servicebio, Wuhan, China). After centrifugation for 10 min (3,000 rpm), the supernatant was used for ELISA (Epoch, BioTeK, Vermont, USA) according to the instructions of the manufacturer.

### 2.8 Metagenomic Sequencing

Extraction and analysis of DNA were performed as previously described [26]. Metagenomic analysis was performed using the DNBSEQ T7 sequencing system (BGI, Shenzhen, China), generating 100 base pair paired‐end sequences for the entire set of samples. After stringent quality refinement with fastp (v0.19.4), applying thresholds such as an average phred score of 20 and a minimum sequence length of 51 base pairs. Host‐associated sequences were removed utilizing Bowtie2 (v2.3.5), ensuring that only high‐quality reads were retained for downstream analysis. Kraken2 and Bracken were used to generate a taxonomic profile and species annotation [33, 34]. The functional analysis was processed by building a genome catalog and further analyses were conducted using MetaWIBELE [35]. The subsequent analysis used eggnog‐mapper to perform database mapping [36]. The Huttenhower Lab Galaxy Server was employed to conduct Linear discriminant analysis Effect Size (LEfSe) to identify the feature that exhibited significant differences between groups [37].

### 2.9 Human Population Cohort Analysis

Metagenomic data was retrieved for 3550 human fecal samples within the Chinese 4D‐SZ cohort [38, 39]. In addition, the Cultivated Genome Reference 2 (CGR2) offers detailed genomic data for 3324 bacterial strains to annotate the 4D‐SZ cohort [26].

### 2.10 Genomic Analysis

The genomic analysis process was performed as described previously [40]. After obtaining the sequence information of *B. virosa* AM16‐14, genome annotation was carried out using Proksee, including identifying and modifying genes and non‐coding regions in the genome to determine the function and characteristics of genes [41]. In addition, functional prediction and annotation of the encoded proteins of *B. virosa* AM16‐14 genome were carried out, including analyses of metabolic pathway, enzyme activity, and protein domains to determine the potential biological impact of encoded proteins.

### 2.11 Untargeted Metabolomics Analysis

A fecal sample (100 mg) was homogenized in a cold mixture of 1 mL methanol‐acetonitrile‐water (2:2:1, v/v), sonicated, and then centrifuged to remove precipitated proteins. The samples were analyzed on a T3 column (ACQUITY UPLC HSS T3 1.8 µm, 2.1 × 100 mm, Waters) coupled to a hybrid quadrupole‐orbitrap mass spectrometer (QExactive, Thermo Scientific) using a UHPLC system (Ultimate 3000, Thermo Scientific) with a mobile phase consisting of acetonitrile (A) and 0.1% formic acid (B) [42]. Each sample was analyzed by FullMS‐ddMS in the positive ion mode.

### 2.12 Targeted Metabolite Detection

Liver and fecal samples were prepared and analyzed according to procedures described previously [43, 44]. Briefly, samples were homogenized in a 0.1% formic acid solution. After freezing and thawing twice with liquid nitrogen, the samples were subjected to water bath shaking (75°C) for 15 min. Internal standards were added for subsequent analysis after ultrasonic extraction. Branched chain amino acids (BCAAs) changes in samples were analyzed on LC‐20A liquid chromatography system (Shimadzu, Japan) equipped with an Intrada Amino Acid column (50 × 3 mm, 3 µm) (Imtakt, USA) coupled to a Sciex 5500 Qtrap mass spectrometer (Sciex, USA), while vitamin B levels in samples were detected using a Kinetex XB C18 column (2.1 × 100 mm, 2.6 µm) (Phenomenex, China). For targeted metabolomics related mobile phases and elution methods, please consult Tables S3 and S4.

### 2.13 Immunofluorescence Analysis

Phospho‐BCKDHA were quantified in periodate, lysine, paraformaldehyde (PLP) buffer‐fixed OCT‐embedded liver samples by immunofluorescence. Tissue slices were incubated with anti‐Phospho‐BCKDHA antibody (1:100) overnight at 4°C and then incubated with fluorophore‐labeled secondary antibody for 1 h at 37°C. Representative images were observed under a positive fluorescence microscope (Nikon Eclipse C1, Japan) and quantification was performed using ImageJ.

### 2.14 Western Blot Analysis

Tissue and cell samples were subjected to protein extraction using pre‐cooled RIPA lysis buffer (Beyotime Biotechnology, Shanghai, China), containing protease and phosphatase inhibitors. After centrifugation, the supernatant was collected, and protein concentration was determined using the BCA assay. Samples were then prepared for electrophoresis by mixing with loading buffer and heating for 10 min at AccuBlock Digital Dry Bath (D1100‐230 V, Labnet International, Inc, USA). Following separation on an SDS‐PAGE gel, the proteins were transferred to a polyvinylidene difluoride (PVDF) membrane. The membranes were blocked with protein‐free fast blocking solution (Servicebio, Wuhan, China) and incubated overnight at 4°C with a 1:2000 diluted primary antibody. After a subsequent 1 h incubation at room temperature with HRP‐conjugated goat anti‐rabbit IgG (H+L) (AS014, ABclonal, Wuhan, China), protein bands were visualized using an enhanced chemiluminescence kit (Beyotime, Shanghai, China) and quantified using ImageJ.

### 2.15 RNA Isolation and RT‐qPCR

Total RNA from fecal samples and liver tissue was isolated using FastPure Cell/Tissue Total RNA Isolation Kit V2 (Vazyme, Nanjing, China) and converted to cDNA (HIScrip II Q RT SUperMix for qPCR, Vazyme, Nanjing, China). RT‐qPCR was performed using ChamQ SYBR Color qPCR Master Mix (Vazyme, Nanjing, China) using an ABI 7500 Real‐time PCR instrument as described previously [45]. Expression data were normalized to the reference gene *GAPDH* and fold‐change was calculated using 2^−∆∆Ct^ method. The primers (Table S5) were synthesized by Sangon Biotech Co., Ltd (Shanghai, China).

### 2.16 Thiamine Analysis of the Population Cohort

Blood samples were collected from a cohort of individuals, including healthy participants (n = 30), patients with mild MASLD (n = 28) and moderate‐severe MASLD (n = 29), based on ultrasound data. In addition, clinical data on body mass index (BMI), fasting levels of blood glucose, LDL, TC, TG, ALT, AST, and thiamine were collected.

### 2.17 Cell Culture Experiments

HepG2 cells were purchased from American Type Culture Collection at December 2024 (HB‐8065, RRID: CVCL_0027, Manassas, Virginia, US). The cells were authenticated by the Institute of Translational Medicine of Zhejiang University before use to confirm that the cells were correct. HepG2 cells were seeded in 6‐well plates (1 × 10^5^ cells/well). After 48 h, the cells were treated with thiamine (50 µm) or thiamine (50 µm) plus Bacimethrin (5 µm). After 6 h of cultivation, cell samples were harvested for further analysis.

### 2.18 Statistical Analysis

The data were presented as mean±SD and analyzed using GraphPad Prism 8.0 software (GraphPad Software, San Diego, Canada). The Shapiro‐Wilk test was used to assess the normality of the data. For normally distributed data, *t*‐test was used to compare two groups, while a one‐way ANOVA is employed for comparisons involving multiple groups, followed by Tukey's multiple comparison test. For non‐normally distributed data, the Wilcoxon rank‐sum test is used for comparisons between two groups, and the Kruskal‐Wallis test was utilized for comparing multiple groups. All the omics data were corrected for false discovery rate (FDR) using the Benjamini‐Hochberg method. *p*<0.05 were considered statistically significant.

## 3 Results

### 3.1 *B. virosa* Is a Key Species Enriched by Administration of STA

**FIGURE 1:**
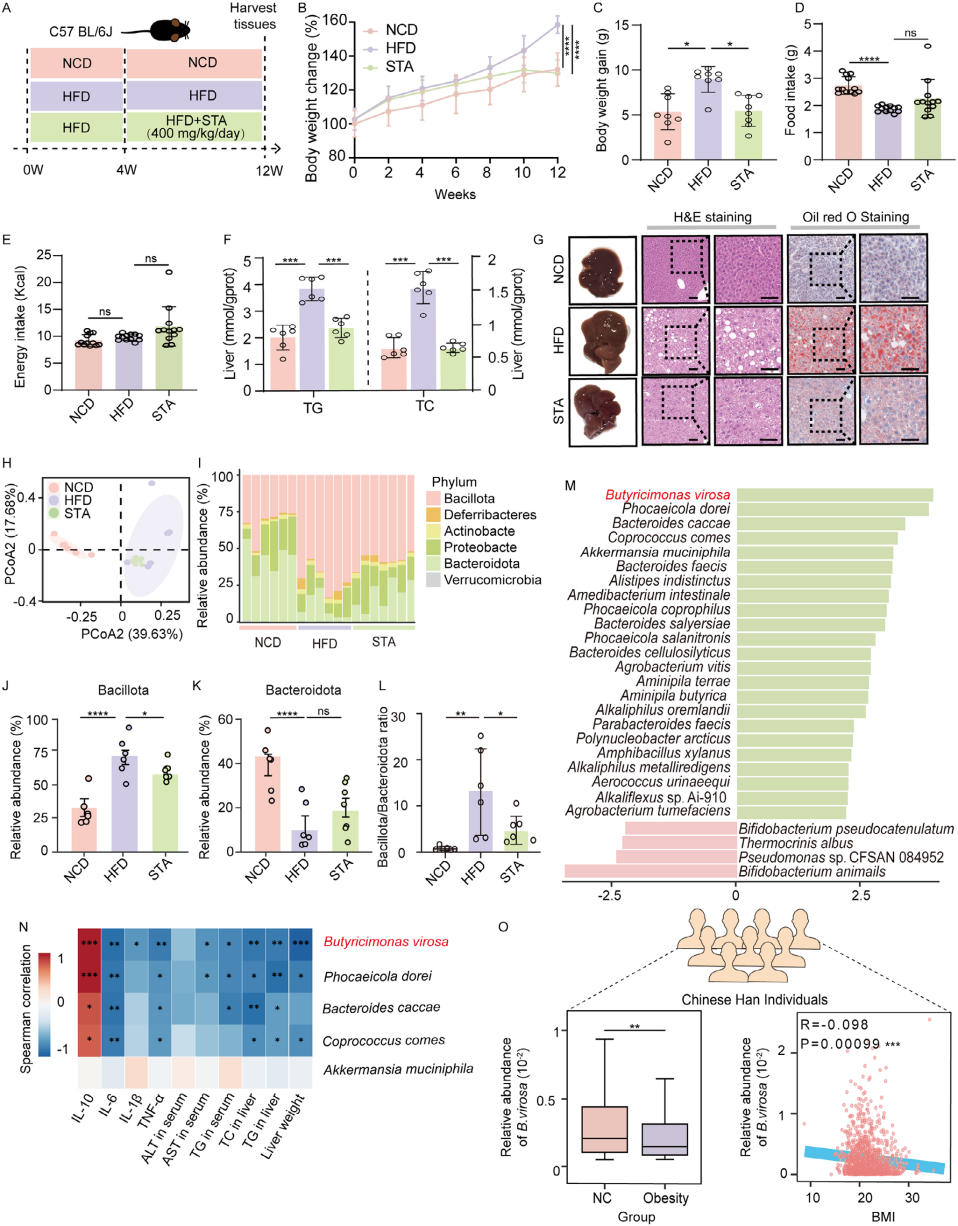
*B. virosa* is a key species affected by STA supplementation. A) Experimental design. n = 8. B) Body weight development. C) Body weight gain. D) Food intake. E) Energy intake. F) Hepatic levels of TG and TC. G) Morphology of the liver, H&E and oil red O staining. Scale bar, 50 µm. H) PCoA clustering of the composition of the gut microbiota in mice in the NCD, HFD, and STA groups. n = 6. I) Phylum‐level distribution for the NCD, HFD, and STA groups. Relative abundance of Bacillota J) and Bacteroidota K) in feces. L) Changes of the Bacillota/Bacteroidota ratio. M) LEfSe analysis of metagenomic changes in response to STA administration. N) Spearman's correlation analysis of STA enriched species and MASLD phenotypes. O) The abundance of *B. virosa* in lean (NC) and obese individuals, and relationship between BMI and *B. virosa* in the 4D‐SZ cohort. n = 3550. The lean is defined as individuals with a BMI < 24 kg/m^2^, and obese refers to those with a BMI ≥ 28 kg/m^2^. Comparison between indicated groups, ^*^ *p<*0.05, ^**^ *p<*0.01, ^***^ *p<*0.001, ^****^ *p<*0.0001, ns: no significance.

Our previous study demonstrated that supplementation with STA counteracted obesity development and modulated the composition of the gut microbiota in mice [46]. To further examine the underlying mechanisms driving the effects of STA administration, we administered a high dose of STA (400 mg/kg/day) to a mouse model of HFD‐feeding‐induced MASLD (Figure 1A). Our results recapitulated our previous finding revealing that compared with mice fed the HFD, mice fed the HFD supplemented with STA exhibited a significant decrease in body weight gain (Figure 1B,C). Additionally, the use of STA did not significantly alter food intake (Figure 1D) and energy consumption (Figure 1E), indicating that weight reduction associated with STA may not occur through appetite suppression or changes in food consumption. We further observed that supplementation with STA resulted in a reduction in the levels of serum TC, TG, and AST, the latter a marker of liver damage (Figure S1A,B). Compared to mice fed the HFD, supplementation with STA significantly reduced liver weight (Figure S1C) and levels of TC and TG in the liver (Figure 1F). STA‐treated mice displayed morphological improvements in liver and a reduction in fat accumulation as revealed by H&E staining (Figure 1G; Figure S1D) and oil red O staining (Figure 1G; Figure S1E), supporting the efficacy of STA in alleviating MASLD. In mice treated with STA, the levels of pro‐inflammatory cytokines (TNF‐α, IL‐1β, and IL‐6) in serum were reduced, while the levels of the anti‐inflammatory cytokine IL‐10 were increased (Figure S1F). These results collectively indicate that STA may not only counteract hepatocyte damage but also effectively reduce lipid burden in the liver, thereby lowering the risk of MASLD progression.

The metagenomic sequencing of fecal samples indicated that STA did not significantly alter the overall composition of the gut microbiota in mice as demonstrated by a PCoA analysis (Figure 1H). Accordingly, we did not observe significant differences in the Shannon index and the Simpson index between the HFD‐fed mice and mice fed the HFD supplemented with STA (Figure S2A,B). Still, further analysis based on operational taxonomic units (OTUs) in the gut microbiota of mice revealed significant differences in microbial community composition and diversity among the different treatment groups (Figure S2C). At the phylum level, STA supplement at least partially reversed the changes in microbiota composition induced by intake of the HFD (Figure 1J,K). The Bacillota/Bacteroidota ratio is acknowledged as an important indicator of gut microbiota composition and MASLD progression. Compared to chow fed mice, mice fed the HFD exhibited a significant increase in the Bacillota/Bacteroidota ratio (*p*<0.01), and STA supplementation significantly decreased the Bacillota/Bacteroidota ratio (*p*<0.05) (Figure 1L). Taken together, these results imply that the anti‐MASLD effect of STA was associated with a modulation of the gut microbiota in HFD‐fed mice supplemented with STA.

LEfSe analysis revealed that *B. virosa*, *Phocaeicola dorei*, *Bacteroides caccae*, *Coprococcus comes*, and *A. muciniphila* were the top five species enriched by STA supplementation (Figure 1M). Previous studies have shown that the level of *Akkermansia* and *Alistipes* may be positively correlated with the production of thiamine [47], so we evaluated the relative abundance of these two genera. The results showed that the addition of STA did not change the relative abundance of these two genera (Figure S2D,E). To further study the correlation between enriched gut bacteria and metabolic phenotypes caused by intake of a HFD, we performed Spearman correlation analysis. As shown in Figure 1N, except for *A. muciniphila*, the other four bacteria were inversed correlated with metabolic features, including liver weight, hepatic TG, TC, and AST. Additionally, *B. virosa* and *P. dorei* exhibited the strongest positive correlation with the level of IL‐10 in serum, while displaying significantly inverse correlations with pro‐inflammatory cytokines (TNF‐α, IL‐1β, and IL‐6) (Figure 1N). Since *B. virosa* exhibited the greatest increase in abundance following administration of STA, we examined the metagenomic dataset of 3550 Han Chinese individuals of the 4D‐SZ cohort [38]. The analysis revealed that the abundance of *B. virosa* was significantly decreased in obese individuals (*p<*0.01) and further that the abundance of *B. virosa* was inversed correlated with BMI (*p<*0.001, Figure 1O).

### 3.2 *B. virosa* Attenuates HFD‐Induced MASLD and Gut Microbiota Dysbiosis in Mice

**FIGURE 2:**
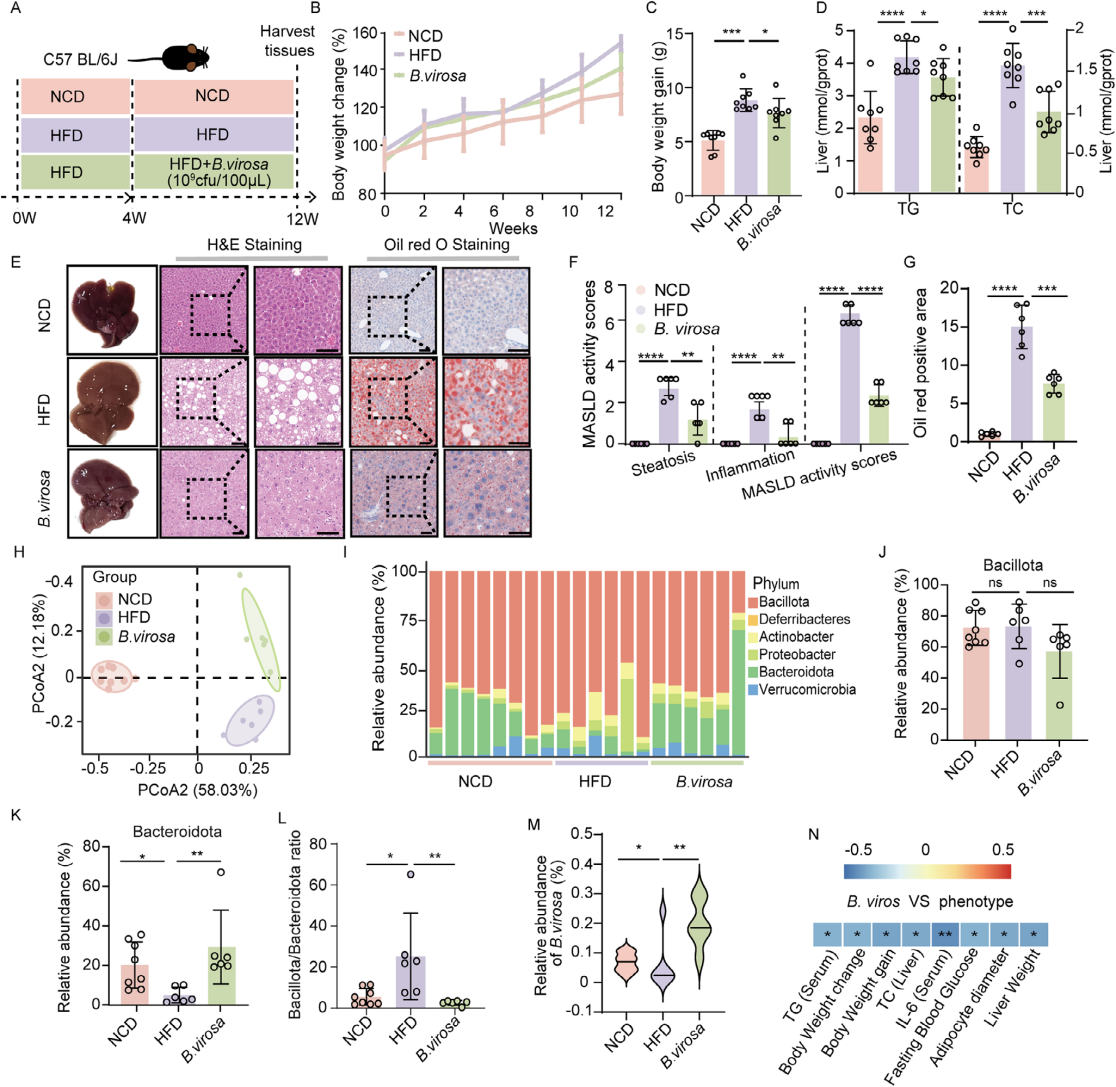
*B. virosa* attenuates HFD‐induced MASLD and reverses HFD induced changes in gut microbiota. A) Experimental design. n = 8. B) Body weight development. C) Body weight gain. D) Hepatic levels of TG and TC. E) The morphology of liver, H&E staining and oil red O staining of liver tissues. Scale bar, 50 µm. F) H&E scores for liver tissues. G) Oil red positive area for liver tissues. H) PCoA clustering diagram of the composition of the gut microbiota in mice in the NCD, HFD, and *B. virosa* groups. n = 6. I) Phylum‐level distribution for the NCD, HFD, and *B. virosa* groups. Relative abundance of Bacillota J) and Bacteroidota K) in feces from the NCD, HFD, and *B. virosa* groups. L) The change of Bacillota/Bacteroidota ratio. M) Relative abundance of *B. virosa* in colon. N) The Spearman's coefficient analysis of correlation between abundance of *B. virosa* and MASLD phenotypes. Comparison between indicated groups, ^*^ *p<*0.05, ^**^ *p<*0.01, ^****^ *p<*0.0001, ns: no significance.

A previous study indicated that *B. virosa* is capable of producing butyric acid and isobutyric acid [13]. However, the physiological functions of *B. virosa* in the mammalian gut remain poorly understood. We examined the effect of *B. virosa* AM16‐14, isolated and identified by BGI, on hepatic steatosis and associated metabolic disorders in an HFD‐induced MASLD mouse model (Figure 2A). Administration of *B. virosa* AM16‐14 significantly reduced body weight gain (Figure 2B,C), fasting insulin (Figure S3A), fasting blood glucose (Figure S3B), and HOMA‐IR (Figure S3C). The *B. virosa*‐treated mice showed a significantly reduced rise in blood glucose levels following glucose loading in comparison to the HFD group (Figure S3D,E), without altering energy intake (Figure S3F,G). The results indicated that continuous administration of *B. virosa* AM16‐14 improved glucose tolerance and insulin sensitivity. In addition, we observed a significant reduction in the size of adipocytes in eWAT (Figure S3H,I). Compared to mice fed the HFD, supplementation with *B. virosa* significantly reduced liver weight (Figure S4A), liver index (Figure S4B), and levels of TC and TG in the liver (Figure 2D). According to the histological evaluation, liver lipid accumulation, inflammatory cell infiltration, and fibrogenesis were significantly reduced after intervention with *B. virosa* compared with HFD fed mice (Figure 2E,F). Thus, oil red O staining indicated that the HFD diet significantly increased the accumulation of fat in the liver, while *B. virosa* treatment reduced fat accumulation (Figure 2E,G). In addition, *B. virosa* treatment reduced serum AST and ALT levels in mice (Figure S4C,D). Furthermore, *B. virosa* treatment decreased the level of the inflammatory cytokines TNF‐α, IL‐1β and IL‐6 in serum and increased the level of the anti‐inflammatory cytokine IL‐10 (Figure S4E). Collectively, these findings suggest that *B. virosa* supplementation could potentially serve as an adjuvant therapy to alleviate MASLD symptoms and ameliorate metabolic abnormalities.

The potential anti‐MASLD mechanism of *B. virosa* was further investigated through metagenomics and metabolomics analyses. Treatment of *B. virosa* modulated the composition of gut microbiota (Figure 2H) and reversed the differences caused by HFD at the phylum level (Figure 2I). At the phylum level, *B. virosa* slightly decreased Bacillota (Figure 2J) and significantly counteracted the HFD‐induced reduction of Bacteroidota (Figure 2K). The reduction of Bacillota/Bacteroidota ratio further indicated that *B. virosa* improved the balance in the intestinal microbiota (Figure 2L). These results suggest a potential role for *B. virosa* in promoting a healthier microbiota, which may help mitigate metabolic disorders associated with HFD‐induced MASLD. Metagenomic analysis also showed a significant increase in the abundance of *B. virosa* in the stool of the mice receiving *B. virosa* compared to mice on the HFD diet alone (*p<*0.01). Since mice were gavaged daily it is not possible in this setting to determine whether *B. virosa* would stably colonize the gut (Figure 2M). The abundance of *B. virosa* was inversed correlated with levels of liver TC and serum TG, body weight, serum IL‐6, fasting blood glucose, adipocyte diameter, and liver weight in the MASLD model (Figure 2N). In conclusion, *B. virosa* supplementation seems to hold promises as an adjunct therapy to potentially mitigate metabolic disorders associated with HFD‐induced MASLD by regulating intestinal microbiota composition and intestinal metabolites.

### 3.3 Anti‐MASLD Effects of *B. virosa* via Gut Microbiota‐Derived TMP Metabolism

**FIGURE 3:**
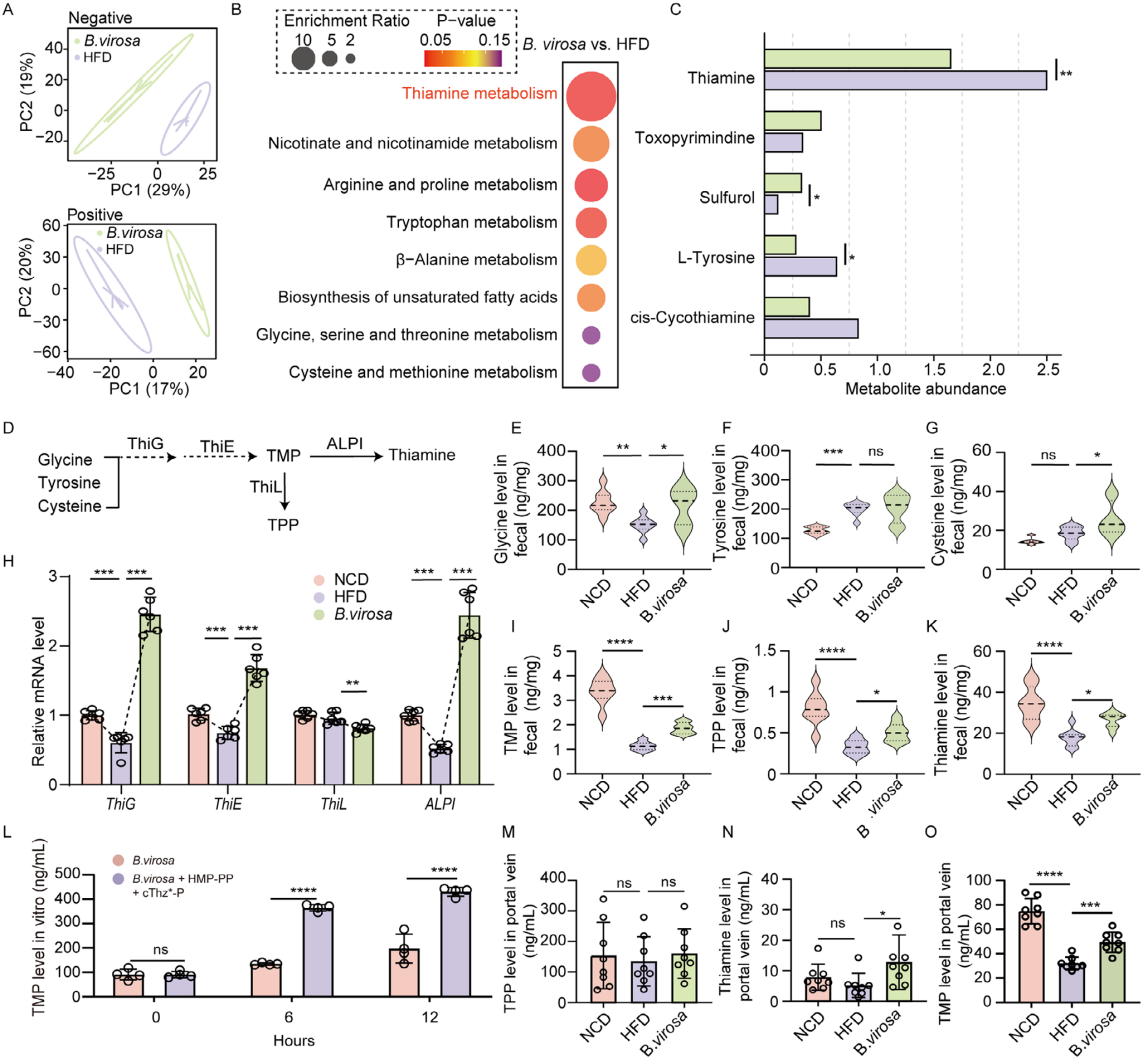
*B. virosa* intervention alters bacterial thiamine synthesis. A) PCoA clustering diagram for metabolites in fecal samples. n = 5. B) Enrichment analysis of differentially affected metabolites. C) The relative content of metabolites associated with thiamine metabolism. D) The metabolic pathway from amino acids to TMP, The level of glycine E), tyrosine F) and cysteine G) in fecal samples. H) Expression of thiamine metabolism related genes The level of TMP I), TPP J) and thiamine K) in fecal samples. L) Changes in TMP content synthesized by *B. virosa* in vitro. The level of TMP M), TPP N) and thiamine O) in hepatic portal vein. Comparison between indicated groups, ^*^ *p<*0.05, ^**^ *p<*0.01, ^***^ *p<*0.001, ^****^ *p<*0.0001, ns: no significance.

To further investigate the mechanisms by which *B. virosa* alleviates MASLD, we conducted both untargeted and targeted metabolomic analyses. As shown in Figure 3A, treatment of *B. virosa* significantly altered the composition of metabolites in fecal samples. Furthermore, enrichment analysis revealed that pathways involved in thiamine metabolism exhibited the most marked changes in response to supplementation with *B. virosa* (Figure 3B). Further examination of thiamine‐related metabolites showed that thiamine and L‐tyrosine levels increased significantly, while sulfurol levels significantly decreased following *B. virosa* treatment (Figure 3C). In addition to L‐tyrosine, we observed significant changes in metabolic pathways related to multiple amino acids, including arginine, proline, alanine, and glycine (Figure 3B). Amino acids serve as crucial precursor molecules for thiamine synthesis. Among them, glycine, tyrosine, and cysteine are the most important amino acids involved in this process (Figure 3D) [48]. Subsequently, we carried out a targeted detection of the contents of these three amino acids. The results showed that the *B. virosa* treatment significantly increased the levels of glycine and cysteine (*p*<0.05) (Figure 3E–G). According to the thiamine biosynthesis pathway in prokaryotes [49, 50], the metabolic processes that convert amino acids into thiamine require the prior synthesis of TMP (Figure 3D). *B. virosa* treatment significantly up‐regulated the mRNA levels of bacterial thiamine metabolism‐related genes in the gut microbiota community as determined by RT‐qPCR (Figure 3H). Specifically, expression of the bacterial genes *ThiG*, *ThiE*, and *ALPI* were significantly up‐regulated following treatment with *B. virosa* (*p*<0.001). These genes are involved in the biosynthesis of TMP and thiamine. In contrast, the expression of *ThiL* was significantly down‐regulated (*p*<0.01), a key enzyme responsible for catalyzing the conversion of TMP into TPP. To further quantify the levels of thiamine‐related metabolites, targeted metabolomics analysis was performed, measuring the levels of TMP, TPP, and thiamine in fecal samples. Following *B. virosa* treatment, we observed an increase in TMP (*p*<0.001), TPP (*p*<0.05), and thiamine (*p*<0.05) levels in feces (Figure 3I–K). In addition, providing the necessary precursors for thiamine synthesis to *B. virosa* significantly increased the content of TMP in the culture medium after cultivation (Figure 3L). To determine whether TMP derived from *B. virosa* directly enters the liver through enterohepatic circulation, the levels of TMP, TPP and thiamine in the portal vein were detected (Figure 3M–O). The results showed significantly higher TMP levels in the portal vein of the *B. virosa* group than in the HFD group (*p*<0.001), confirming a transport pathway for TMP from the gut to the liver. These results suggest that *B. virosa* enhances the availability of bacterially derived thiamine, which may contribute to the beneficial effects of administration of *B. virosa* in alleviating steatotic fatty liver conditions.

### 3.4 *B. virosa* Facilates BCAAs Degradation Through Gut Microbiota‐Derived Thiamine

**FIGURE 4:**
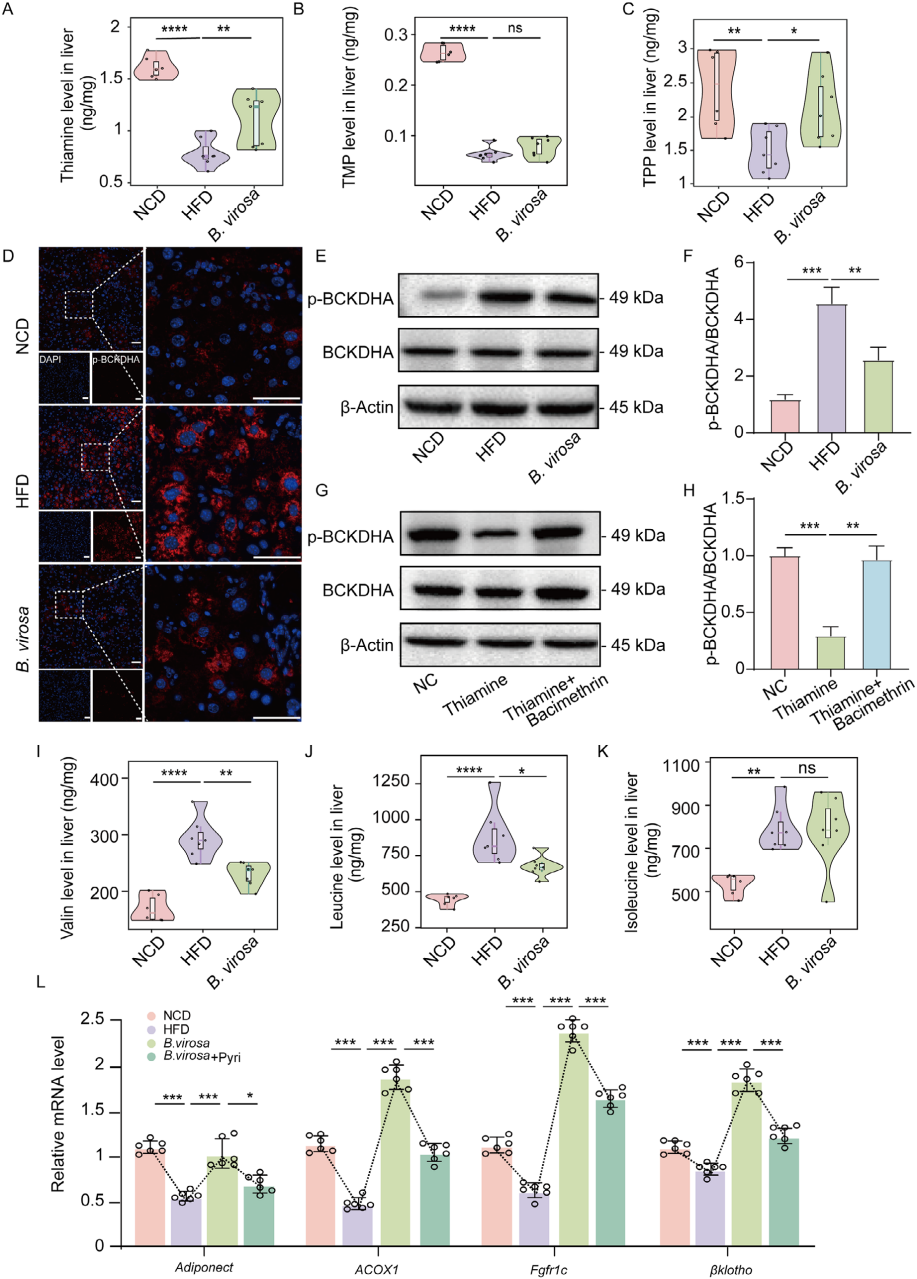
Effects of *B. virosa* on TPP‐induced BCAAs degradation in liver. Hepatic thiamine A), TMP B), and TPP C) levels. After grinding the liver samples, targeted metabolite analysis was performed. n = 7. D) Representative immunofluorescence images of liver sections stained with phosphorylated BCKDHA (p‐BCKDHA). E) Levels of p‐BCKDHA and BCKDHA in liver samples using western blot. F) Quantitative ratio analysis of protein expression. G) Levels of p‐BCKDHA and BCKDHA in HepG2 cells. H) Quantitative ratio analysis of protein expression. n = 3. Hepatic valine I), leucine J) and isoleucine K) levels. L) Expression mRNA levels of genes relevant to lipid metabolism. n = 7. Comparison between indicated groups, ^*^ *p<*0.05, ^**^ *p<*0.01, ^***^ *p*<0.001, ^****^ *p<*0.0001, ns: no significance.

To investigate whether increased thiamine levels in gut resulted in alterations of thiamine content and lipid metabolism in the liver, we measured thiamine, TMP, and TPP levels in the liver. As shown in Figure 4A–C, HFD‐feeding significantly reduced the levels of hepatic thiamine (*p*<0.0001), TMP (*p*<0.0001), and TPP (*p*<0.01), while *B. virosa* treatment significantly increased the concentration of hepatic thiamine (*p*<0.01) and TPP (*p*<0.05).

BCAAs have been shown to exert diverse metabolic effects and have been associated with insulin resistance and other metabolic disorders [51]. The degradation of BCAAs is regulated by the branched‐chain α‐keto acid dehydrogenase E1 subunit α (BCKDHA) and TPP serves as a coenzyme that is essential for the function of the E1 component in the BCKDH complex, facilitating the dephosphorylation and activation of BCKDHA [52]. As shown in Figure 4D–F, the level of p‐BCKDHA was significantly increased in the liver of MASLD mice, and *B. virosa* intervention resulted in a decrease in the phosphorylation level of BCKDHA. In the colon, TMP is typically taken up directly by intestinal epithelial cells and utilized for metabolic processes. Thiamine can further be transported to the liver via the portal vein, where it is subsequently converted into TPP [22, 23]. In vitro analysis performed in HepG2 cells demonstrated that thiamine treatment enhances the dephosphorylation of BCKDHA. Conversely, the addition of Bacimethrin, which blocks the conversion of thiamine to TPP, inhibits this dephosphorylation process (Figure 4G,H). Thus, increased levels of hepatic TTP may result in an increased dephosphorylation of BCKDHA leading to an increase in the degradation of BCAAs. Accordingly, we found that HFD elevated the levels of BCAAs in the liver (Figure 4I–K), whereas *B. virosa* significantly reduced the levels of valine (*p*<0.01) and leucine (*p*<0.05). Therefore, these results suggest that gut microbiota‐derived thiamine metabolism may play a role by regulating hepatic TPP levels and BCKDHA activity leading to enhanced metabolism of BCAAs. Finally, we measured the expression of genes playing important roles in metabolic regulation in liver. This analysis showed that expression of *Adiponect, ACOX1, Fgfr1c*, and *βklotho*, was significantly decreased in response to intake of the HFD but increased significantly following intervention with *B. virosa* AM16‐14 (Figure 4L). Thus, administration of *B. virosa* may via increased hepatic levels of TTP enhance degradation of BCAAs improving various metabolic parameters, and in addition exert beneficial effects in relation to increased expression of genes involved in lipid catabolism and FGF21‐related signaling.

To further corroborate the link between administration of *B. virosa* and hepati thiamin levels and metabolism, we used a structural analogue of thiamine, pyridamine (Pyri), which functions as a thiamine antagonist to competitively inhibit the absorption and utilization of thiamine (Figure S5A). *B. virosa* supplementation partially restored the fecal levels of TMP, TPP, and thiamine, and co‐treatment with Pyri did not prevent this restoration regarding TMP and TPP, whereas a slight reduction in thiamine level was observed in the *B. virosa* plus Pyri treated mice. These finding suggest that administration of Pyri did not significantly modulate intestinal thiamine absorption (Figure S5B–D). Of note, Pyri co‐administration abolished *B. virosa*‐induced elevation of hepatic TPP and thiamine, confirming its ability to specifically inhibit hepatic TPP generation or retention (Figure S5E–G). HFD increased body weight (Figure S5H) and liver weight (Figure S5I) relative to NCD, effects attenuated by *B. virosa*. Pyri co‐treatment partially prevented these improvements, leading to higher body and liver weights compared to the *B. virosa* group (Figure S5K). White adipose tissue weight was unaffected by Pyri (Figure S5J). Compared with the *B. virosa* group, mice treated with *B. virosa* in combination with Pyri showed a significant increase in blood glucose levels after glucose loading (Figure S5L–N). The increased levels of *Adiponect*, *ACOX1*, *Fgfr1c*, and *βklotho* mRNAs in response to *B. virosa* administration returned to HFD levels by co‐administration of Pyri (Figure 4L).

These results indicate that thiamine potentially synthesized by the gut microbiota is converted into TPP in the liver where it regulates metabolism of both BCAAs and lipids. highlighting the impact of TPP accumulation associated with *B. virosa* supplementation on these metabolic processes.

### 3.5 Beneficial Effects of *B. virosa* Under Thiamine‐Deficient Conditions

**FIGURE 5:**
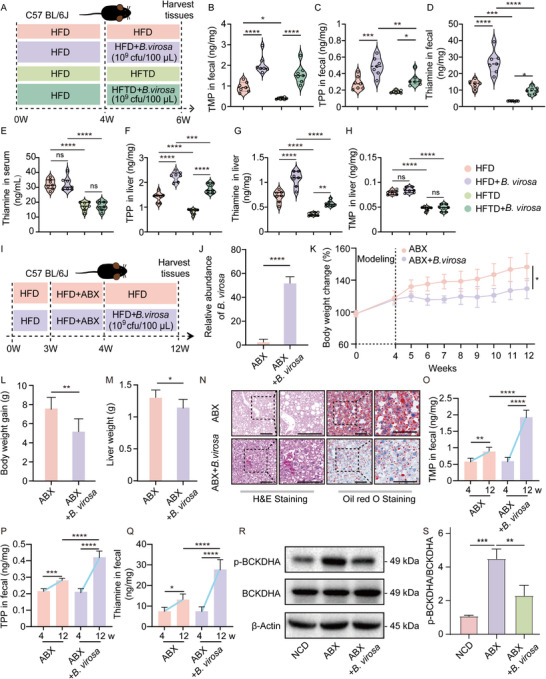
Effects of *B. virosa* on hepatic thiamine levels and BCAAs metabolism in thiamine‐deficient conditions. A) Experimental design using an HFTD diet model. n = 7. The level of TMP B), TPP C) and thiamine D) in fecal samples. E) Serum thiamine levels. Hepatic TPP F), thiamine G) and TMP H) levels. I) Experimental design using ABX treated model. n = 6. J) Relative abundance of *B. virosa* in fecal samples. K) The body weight change. L) The final body weight gain. M) Liver mass. N) H&E staining and Oil Red O staining of liver tissues. TMP O), TPP P) and thiamine Q) levels in liver. R) Western blot analysis for p‐BCKDHA and BCKDHA. S) Quantitative analysis. Comparison between indicated groups, ^*^ *P*<0.05, ^**^ *P*<0.01, ^***^ *p*<0.001, ^****^ *p*<0.0001.

To examine the importance of TPP in the liver, we conducted *B. virosa* gavage treatment alongside intake of a high‐fat thiamine‐deficient (HFTD) diet (Figure 5A). Given that HFTD‐feeding can induce substantial neurological damage in mice, under normal experimental conditions, a two‐week HFTD dietary intervention is typically implemented [53]. Two weeks of HFTD treatment significantly reduced levels of fecal TMP (Figure 5B, *p*<0.05), TPP (Figure 5C), and thiamine (Figure 5D, *p*<0.001). By contrast, *B. virosa* treatment increased fecal TMP (Figure 5B, *p*<0.0001), TPP (Figure 5C, *p*<0.05), and thiamine levels (Figure 5D, *p*<0.05). We further examined serum thiamine content and discovered that HFTD‐feeding significantly decreased serum thiamine levels. Treatment of *B. virosa* did not significantly increase the concentration of thiamine in serum (Figure 5E). This may be due to the relatively short duration of gavage with *B. virosa* but could also be due to passage through the liver functioning as a metabolic sink. However, the levels of hepatic TPP (Figure 5F, *p*<0.0001) and thiamine (Figure 5G, *p*<0.0001) were significantly increased after *B. virosa* treatment. Still, the level of hepatic TMP (Figure 5H) did not show a statistically significant change after *B. virosa* supplementation. Therefore, these results suggest that gut microbiota‐derived thiamine may play a role in restoring levels of thiamine and its metabolites, potentially contributing to the protective effects against MASLD.

To further examine the potential role of *B. virosa*, we performed *B. virosa* treatment after administering a cocktail of antibiotics (ABX) (Figure 5I). Compared with the ABX group, the number of *B.virosa* in the feces of mice treated with ABX significantly increased (*p*<0.001) (Figure 5J). Following ABX treatment, *B. virosa* was still able to reduce HFD‐induced increases in body weight (Figure 5K), body weight gain (Figure 5L, *p*<0.01), liver mass (Figure 5M, *p*<0.05), and eWAT mass (Figure S6A, *p*<0.05). Additionally, *B. virosa* treatment decreased the characteristic features of MASLD (Figure 5N; Figure S6B) and reduced hepatic fat accumulation (Figure S6C). Furthermore, *B. virosa* significantly increased the levels of TMP (Figure 5O, *p*<0.0001), TPP (Figure 5P, *p*<0.0001), and thiamine (Figure 5Q, *p*<0.0001). When analyzing the levels of phosphorylated BCKDHA (p‐BCKDHA) in the liver, we found that *B. virosa* treatment still enhanced the dephosphorylation of BCKDHA even after ABX treatment (Figure 5R,S). This indicates that the beneficial effects of *B. virosa* on modulating liver metabolism and mitigating the effects of HFD remain intact despite antibiotic intervention, suggesting that *B. virosa* plays a critical role in regulating thiamine levels and influencing the phosphorylation status of key metabolic enzymes involved in BCAAs degradation.

### 3.6 Population Cohort Corroboration of the Correlation Between Thiamine and MASLD

**FIGURE 6:**
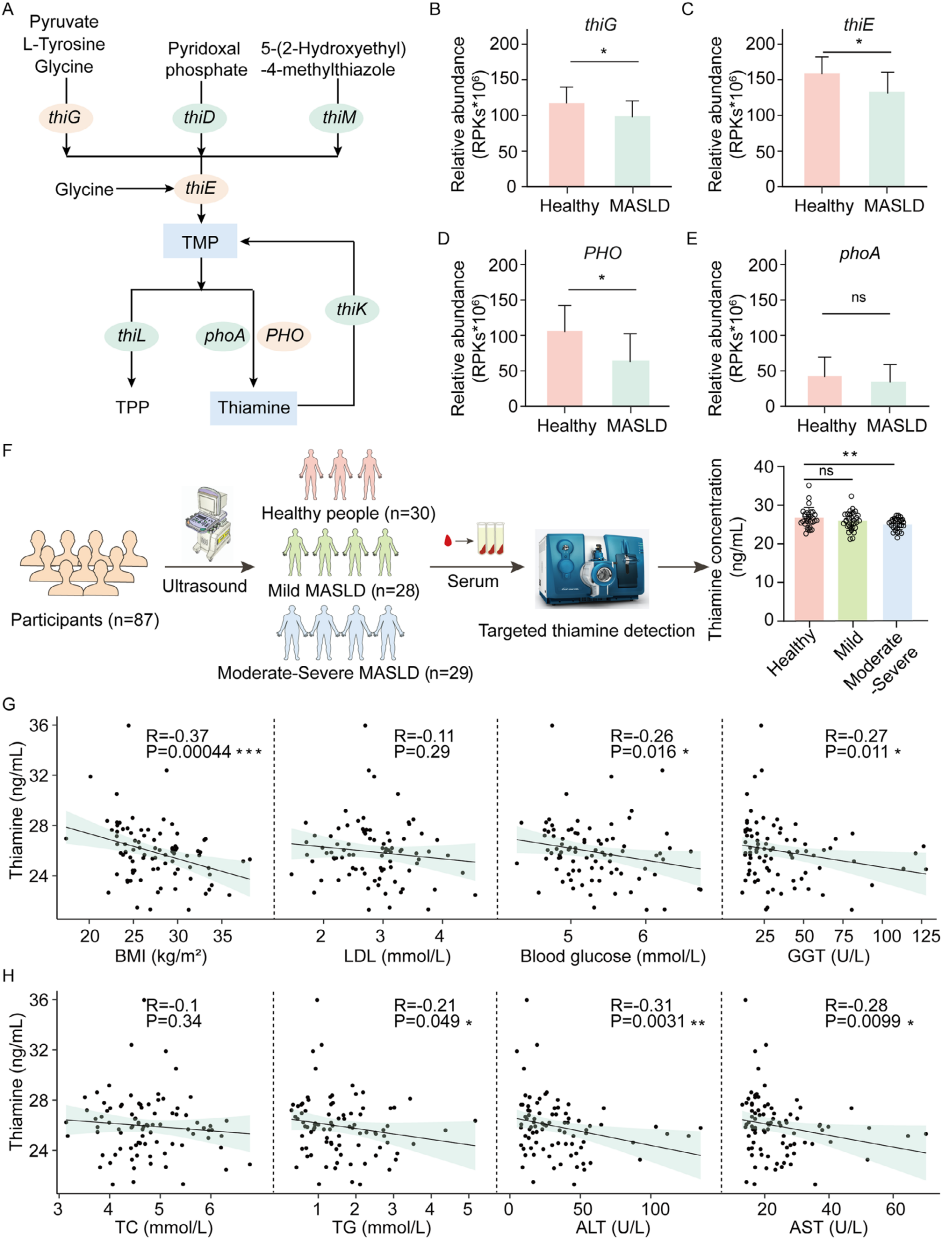
Population cohort analysis of the correlation between thiamine and clinical indicators of MASLD. A) Thiamine related metabolism. The abundance of *ThiG* B), *ThiE* C), *PHO* D) and *phoA* E) using public population cohort. Healthy: n = 20, MASLD: n = 22. F) Analysis process of population cohort and measurement of thiamine in blood samples. Correlation of BMI/LDL/blood glucose/GGT G) and TC/TG/ALT/AST H) with blood thiamine level. Comparison between indicated groups, ^*^ *p*<0.05, ^**^ *p*<0.01, ns: no significance.

To investigate the relationship between thiamine metabolism and MASLD in a population cohort, we quantitatively analyzed the gene abundance of key enzymes involved in thiamine synthesis in the gut microbiota. Via the catalytic action of the enzymes ThiG and ThiE, amino acids are converted into TMP (Figure 6A). Subsequently, TMP is further transformed into thiamine through the sequential actions of *phoA* and PHO [54]. In patients with MASLD, the gene abundances of *ThiG* (Figure 6B, *p*<0.05), *ThiE* (Figure 6C, *p*<0.05), and *PHO* (Figure 6D, *p*<0.05) were significantly decreased, while there was no change in the levels of *phoA* (Figure 6E; Figure S7). The reduction of these key enzymes in thiamine metabolism may serve as a critical risk factor in the progression of MASLD and represents a potential intervention target.

To further substantiate the association between thiamine levels in blood and MASLD, a total of 87 serum samples were collected from a cohort of individuals, comprising healthy participants (n = 30) and patients with mild (n = 28) and moderate‐severe (n = 29) MASLD, as determined by ultrasound data (Figure 6F). Targeted analysis of these samples revealed a significant inverse correlation between thiamine concentration with the severity of MASLD (Figure 6F, *p*<0.01). Further assessments were performed to investigate the correlation between thiamine concentrations and various clinical indicators (Figure 6G,H). The results revealed a significant inverse correlation between thiamine levels and BMI (*p*<0.001), fasting blood glucose (*p*<0.05), GGT (*p*<0.05), TG (*p*<0.05), ALT (*p*<0.01), and AST (*p*<0.05). These results suggest that lower thiamine levels are associated with increased liver injury and dysfunction in glucose‐lipid metabolism. In conclusion, we established a link between thiamine levels and key metabolic and liver‐related parameters within the studied population, providing further support for the notion that thiamine may play an important role in the pathophysiology of MASLD.

**FIGURE 7:**
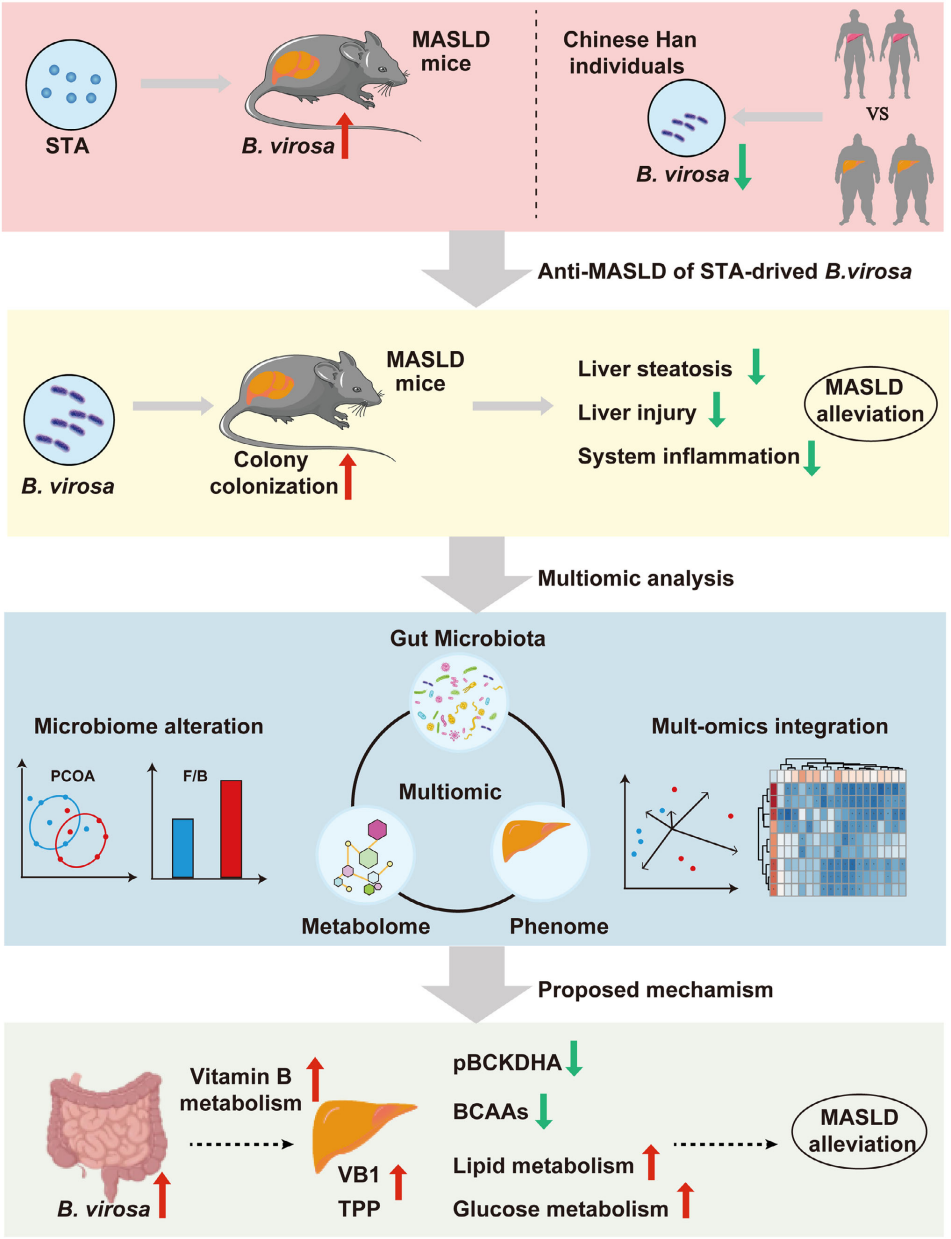
A proposed model summarizing how *B. virosa* modulates gut microbiota‐dependent thiamine metabolism and attenuates mouse steatotic liver disease. Solid lines and filled symbols represent established findings supported by experimental data; dashed lines and open symbols represent hypothetical mechanisms requiring further validation.

In summary, STA‐induced *B. virosa* colonization in NAFLD mice, through multi‐omics integrated analysis, improved hepatic steatosis, injury, and systemic inflammation by regulating VB1/TPP and BCAA metabolism, thereby alleviating NAFLD (Figure 7).

## 4 Discussion

MASLD represents a significant global health challenge, necessitating innovative therapeutic strategies. Recent advances in the use of prebiotics and probiotics offer promising avenues for intervention by modulating gut microbiota composition and function [55, 56]. These strategies based on the regulation of the gut microbiota can enhance the production of beneficial metabolites, such as short‐chain fatty acids, vitamins, amino acids, and secondary metabolites, which may ameliorate the pathological processes underlying MASLD [57, 58]. Targeting the gut‐liver axis through these dietary interventions not only addresses liver health but also presents a multifaceted approach to improving overall metabolic function, emphasizing the potential of microbiota‐targeted therapies in chronic liver disease management [59, 60]. In this study, we present evidence demonstrating that STA functions as a prebiotic and identify *B. virosa* as a key bacterium inhibiting MASLD progression. This study elucidates potential mechanisms by which *B. virosa* alleviates MASLD and provides new insights into the potential importance of gut microbiota‐derived thiamine metabolism. We further describe a significant correlation between thiamine deficiency and the progression of MASLD indicating that modulation of the gut microbiota to enhance thiamine synthesis may serve as a promising strategy.

STA is recognized as a prebiotic that modulates the gut microbiota, thereby contributing to the prevention and management of various diseases [6]. This study further extends the application of STA in the prevention and treatment of MASLD, identifying key bacterial strains and exploring potential mechanisms. Our results support the critical role of *B. virosa* as a gut bacterium with potential impact on MASLD development in mice and report on a significant inverse correlation between the abundance of *B. virosa* and BMI in a human population cohort analysis.

Although the genus of *Butyricimonas* have been associated with metabolic health and longevity [61], detailed information of mechanisms governing the probiotic effects remain scarce. Herein, we demonstrate that gavage of *B. virosa* alleviated the development and symptoms of MASLD (Figure 2). To further examine the potential mechanism by which *B. virosa* alleviates MASLD, we conducted an in‐depth investigation integrating metagenomics and metabolomics analyses. Sakamoto et al. have shown that *B. virosa* is capable of producing butyric acid and isobutyric acid [13]. However, our analysis indicates that most significant alterations are observed in relation to thiamine metabolism (Figure 3).

Humans predominantly obtain thiamine through dietary intake, and thiamine deficiencies can lead to detrimental neurological effects [62]. In addition to obtaining thiamine from the diet, the gut microbiota also possesses the capability to synthesize TMP and subsequently convert it into other active forms [63]. However, knowledge of the role of prebiotics and probiotics in regulating gut microbiota‐dependent thiamine availability remains limited, and only little information on the impact of gut microbiota‐derived thiamine on MASLD is available. Our findings demonstrate that *B. virosa* treatment significantly increased the fecal concentrations of TMP and thiamine, thereby enhancing hepatic thiamine and TPP content (Figures 3, 4). This subsequently stimulates the activation of the BCKDH complex promoting degradation of BCAAs (Figure 4). Importantly, by measuring Vit B metabolites in the portal vein, we provide evidence for a transport of TMP from the gut to the liver. Taken together, this suggests that modulating the gut microbiota to enhance thiamine accumulation in the liver could be a potentially viable strategy for alleviating MASLD.

Considering that exposure to long‐term HFTD can induce physiological dysfunction in mice, we conducted a two‐week HFTD intervention experiment (Figure 5). The results demonstrated that *B. virosa* could significantly enhance fecal TMP levels in mice fed a HFTD. Further, after treating with ABX for one week to reduce the gut microbiota communities, gavage with *B. virosa* significantly increased the level of TMP in feces and at the same time increased the content of TPP in the liver, thereby activating the activity of the BCKDH complex. These results indicate that *B. virosa* can play a role in regulating liver levels of thiamine and its metabolites and thereby development of MASLD. Future studies will optimize these strategies to further confirm the mediating role of TMP in the protection against MASLD.

Dysbiosis of the gut microbiota, frequently observed in MASLD patients [64], may result in a reduced capacity for thiamine synthesis within the gastrointestinal tract. This internal deficiency risk is compounded because MASLD is a complex metabolic disorder that inherently increases the body's thiamine demands [65, 66]. This heightened demand is compounded in special operational groups, who not only face restricted dietary thiamine access, but whose high energy expenditure accelerates thiamine consumption [19]. Our study revealed that in patients with MASLD, the abundance of key genes associated with the thiamine synthesis and metabolism was significantly decreased (Figure 6). We further observed that during the progression of MASLD, plasma thiamine concentrations significantly decreased, and we observed a inverse correlation between plasma thiamine levels and phenotypes associated with multiple metabolic diseases. These findings suggest that key genes involved in fecal thiamine synthesis, as well as serum thiamine degradation, may potentially serve as biomarkers for the diagnosis of MASLD. In addition, such analyses also provide potential markers for early risk assessment and nutritional intervention in population with high energy consumption and high metabolic load.

In summary, our findings establish the role of prebiotic STA supplementation in alleviating MASLD. Specifically, dietary STA enhances the abundance of *B. virosa* in the gut microbiota. *B. virosa* promotes the synthesis of TMP in the gut, which is subsequently converted into thiamine. Through the enterohepatic circulation, thiamine accumulates in the liver, where it is further converted to TPP. This process enhances the degradation of BCAAs and further modulates hepatic lipid metabolism alleviating the progression of MASLD. Notably, gavage with *B. virosa* effectively ameliorates hepatic TPP deficiency induced by inadequate dietary intake. We also elucidated the mechanism by which gut microbiota‐derived TMP reaches the liver via the portal vein to exert anti‐MASLD effects, providing novel insights into probiotic intervention strategies.

### 4.1 Limitations of the Study

This study has several limitations. First, although we observed a significant inverse correlation between thiamine levels in the blood and the clinical manifestations of MASLD, no significant changes in blood thiamine were observed in the animal experiment. The effect of single prebiotic or probiotic treatment on systemic thiamine concentration was relatively small. This indicates that the regulation of thiamine levels may require complex interactions between the gut microbiota and the host rather than the action of a single strain. Second, how *B. virosa* promotes the synthesis of thiamine and its conversion into TMP and TPP, as well as the specific metabolic pathways, still require further study. Further studies using different preclinical models such as models of non‐alcoholic steatohepatitis (NASH) caused by high‐fructose and high‐fat diets are crucial for a comprehensive assessment of the impact of thiamine regulation on diseases related to metabolic dysfunction. Furthermore, although this study pointed to the potential role of thiamine in liver lipid metabolism, further research is needed to clarify the complex mechanisms behind modulation of lipid metabolism and the overall implication for liver health.

## Author Contributions

N.H. contributed to conceptualization, formal analysis, funding acquisition, visualization, investigation, and writing – original draft, H.W. contributed to formal analysis, visualization, investigation, data curation, methodology, and writing – original draft, Z.Y. contributed to formal analysis, visualization, investigation, data curation, and writing – original draft, H.L. contributed to investigation, resources, and methodology, B.L. contributed to investigation, resources, and methodology, K.C. contributed to formal analysis and investigation, Z.W. contributed to investigation, X.Z. contributed to formal analysis and investigation, H.L. contributed to conceptualization, M.W. contributed to formal analysis, X.L. contributed to conceptualization, Y.Z. contributed to formal analysis, H.Z. contributed to conceptualization, L.X. contributed to conceptualization, K.K. contributed to data curation, project administration, and writing – review and editing, J.P. contributed to data curation, project administration, and writing – review and editing, Y.Z. contributed to conceptualization, funding acquisition, project administration, and writing – review and editing, and S.L. contributed to conceptualization, funding acquisition, project administration, and writing – review and editing.

## Funding

This study was supported by National Natural Science Foundation of China (82470615), Shandong Provincial Youth Entrepreneurship Program for Colleges and Universities (2024KJJ042), Shandong Provincial Natural Science Foundation (ZR2022MH217), the Central Public‐interest Scientific Institution Basal Research Fund, CAFS (2023TD52, 2023TD76), the Shenzhen Municipal Government of China (No. KCXFZ20240903094006009 and JCYJ20241202124801003), the Qingdao Municipal Demonstration Project for Science & Technology to Benefit the People (No.25‐1‐5‐smjk‐13‐nsh), and the National Key Research and Development Program of China (2025YFA1310200).

## Conflicts of Interest

The authors declare no conflicts of interest.

## Supporting information




**Supporting File**: advs73864‐sup‐0001‐SuppMat.docx.

## Data Availability

The original metagenomic data of this article has been uploaded to China National GeneBank DataBase (CNGBdb) with the serial number CNP0005799. A Chinese cohort study (4D‐SZ) deposited in CNGBdb including 3, 350 human gut metagenome sequencing data and clinical indicators (CNP0000426). CGR2 was uploaded into CNGB Sequence Archive (CNSA) of CNGBdb with accession number CNP0000126 and CNP0001833, and NCBI under the projects PRJNA482748 and PRJNA903559. The public database of NAFLD was downloaded at European Nucleotide Archive (ENA) with accession number PRJEB55534. Non‐targeted metabolomic data is available at MetaboLights (https://www.ebi.ac.uk/metabolights/, MTBLS10432). All data are available in the main text, extended data or supplementary materials.
