# Supplementary material for: The Gut Commensal Butyricimonas Virosa Modulates Gut Microbiota‐Dependent Thiamine Metabolism and Attenuates Mouse Steatotic Liver Disease
Source: Adv Sci (Weinh). 2026 Jan 20;13(17):e17596. doi: 10.1002/advs.202517596 (PMC13042768; doi:10.1002/advs.202517596)
Supplement: Supplementary file 1 — Supporting File: advs73864‐sup‐0001‐SuppMat.docx. [file ADVS-13-e17596-s001.docx]

**The Gut Commensal *Butyricimonas virosa* Modulates Gut Microbiota-dependent Thiamine Metabolism and Attenuates Mouse Steatotic Liver Disease**

Ningning He^1#^, Haoyu Wang^1,2,4#^, Zizhen Yang^1#^, Hui Li^1^, Bei Liu^1^, Kaiwei Chen^1^, Zhinan Wu^4,6^, Xinnan Zhao^3^, Hewei Liang^6^, Mengmeng Wang^4,6^, Xiaofang Li^6^, Yiyi Zhong^7^, Haifeng Zhang^7^, Liang Xiao^2,7^, Karsten Kristiansen^5,6*^, Jixing Peng^3*^, Yuanqiang Zou^2,7*^, Shangyong Li^1*^

^1^ School of Basic Medicine, Qingdao Medical College, Qingdao University, Qingdao 266071, China.

^2^ State Key Laboratory of Genome and Multi-omics Technologies, BGI Research, Shenzhen 518083, China.

^3^ Yellow Sea Fisheries Research Institute, Chinese Academy of Fishery Sciences, Qingdao 266071, China.

^4^ College of Life Sciences, University of Chinese Academy of Sciences, Beijing 100049, China,

^5^ Laboratory of Integrative Biomedicine, Department of Biology, University of Copenhagen, Copenhagen, Denmark

^6^ BGI Research, Shenzhen 518083, China.

^7^ BGI Precision Nutrition, Shenzhen 518083, China

Corresponding author: Shangyong Li: lisy@qdu.edu.cn

Yuanqiang Zou: zouyuanqiang@genomics.cn

Jixing Peng: [pengjx@ysfri.ac.cn](mailto:pengjx@ysfri.ac.cn)

[Karsten Kristiansen: kk@bio.ku.dk](mailto:pengjx@ysfri.ac.cn)**SUPPLEMENTARY METHODS**

**Materials**

The normal chow diet (NCD) and HFD (D12492) were purchased from Shandong Boqiu Medical Technology Co., Ltd (Zaozhuang, China), and the detailed composition of the diets is presented in **Table S1**. HFD-Thiamine deficient diet (#HF60-0VB1, HFTD) was purchased from Dyets (Anhui, China). C57BL/6J mice (male, 6 weeks old, 16-18 g) were obtained from Jinan Pengyue laboratory animal breeding Co., Ltd (Jinan, China). The Elisa kits for alanine amiotransferase (ALT, C009-2-1), aspartate aminotransferase (AST, C010-2-1), triglyceride (TG, A110-1-1) and total cholesterol (TC, A111-1-1) were obtained from Nanjing Jiancheng Bioengineering Research Institute (Nanjing, China). TG assay kit (S03027), TC assay kit (S03042), ALT assay kit (S03030) and AST assay kit (S03040) for serum biochemical analysis were purchased from Rayto Life and Analytical Sciences Co., Ltd (Shenzhen, China). Standard samples of DL-leucine, DL-isoleucine, DL-valine and thiamine were obtained for Dr. Ehrenstorfer (Augsburg, Germany). TPP (CDAA-210074) was purchased from Shanghai Anpel Laboratory Technologies Inc. (Shanghai, China). Pyrithiamine (hydrobromide) (P912440) was purchased from Macklin (Shanghai, China). The detailed chemical and supply information are shown in **Table S2**. The internal standard (Cell Free Amino Acid Mixture-13C,15N, #767964-1EA) was obtained from Sigma-Aldrich (Saint Louis, MO, USA).

**Animal grouping**

**Experiment 1:** (1) NCD group: 12 weeks of normal diet; (2) HFD group: 12 weeks of HFD diet; (3) STA group: 12 weeks of HFD diet with STA treatment (400 mg/kg/day) by gavage starting from week five.

**Experiment 2:** (1) NCD group: 12 weeks of normal diet; (2) HFD group: 12 weeks of HFD diet; (3) *B. virosa* group: 12 weeks of HFD diet with administration of *B. virosa* AM16-14 (1×10^10^ CFU/mL) by gavage starting from week five.

**Experiment 3:** Mice were feed the HFD diet for 4 weeks and then divided into 4 groups: (1) HFD group: additional 2 weeks of HFD diet; (2) HFD + *B. virosa* group: additional 2 weeks of HFD diet and administration of *B. virosa* AM16-14 (1×10^10^ CFU/mL) by gavage; (3) HFTD group: additional 2 weeks of HFTD diet; (4) HFTD + *B. virosa* group: additional 2 weeks of HFTD diet and administration of *B. virosa* AM16-14 (1×10^10^ CFU/mL) by gavage.

**Experiment 4:** Mice were treated with ampicillin, 200 mg/kg; metronidazole, 200 mg/kg; vancomycin, 100 mg/kg; neomycin, 200 mg/kg (AMX) by oral gavage for 1 week to reduce the load of intestinal bacteria. (1) ABX group: 12 weeks of HFD diet and with ABX treatment in the fourth week; (2) ABX + *B. virosa* group: 12 weeks of HFD diet, with ABX treatment in the fourth week and administration of *B. virosa* AM16-14 (1×10^10^ CFU/mL) by gavage starting from week five.

**Experiment 5:** (1) NCD group: 12 weeks of normal diet; (2) HFD group: 12 weeks of HFD diet; (3) *B. virosa* group: 12 weeks of HFD diet with administration of *B. virosa* AM16-14 (1×10^10^ CFU/mL) by gavage starting from week five. (4) *B. virosa* + pyrithiamine group: 12 weeks of HFD diet with administration of *B. virosa* AM16-14 (1×10^10^CFU/mL) by gavage and administration of pyrithiamine (500 μg/kg/day) by intraperitoneal injection starting from week five.


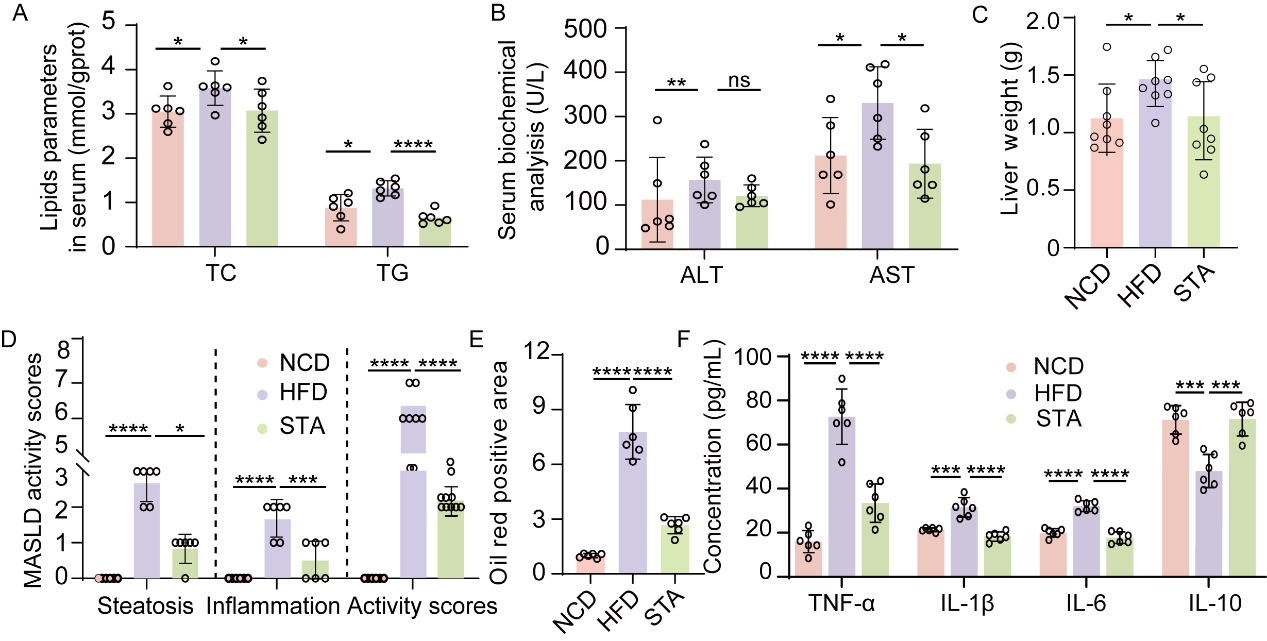


**Figure S1. Impact of STA on MASLD and systemic inflammation.** Biochemical analysis of serum levels of TC and TG (A), ALT and AST (B). (C) Liver weight. (D) MASLD activity scores for liver tissues. (E) Oil red positive area of liver sections. (F) Serum levels of TNF-α, IL-1β, IL-6 and IL-10. Comparison with the HFD group, * *P*<0.05, *** *P*<0.001, **** *P*<0.0001, ns: no significance.


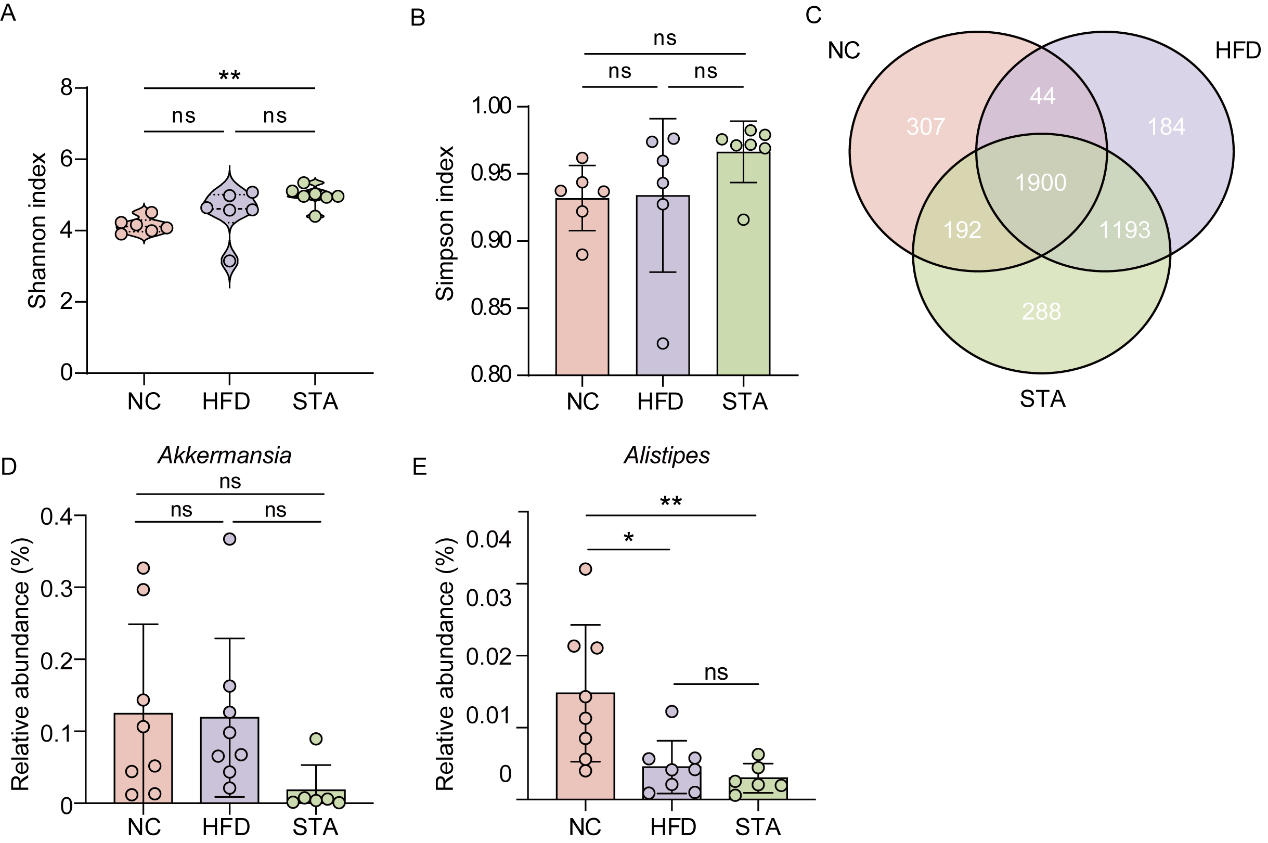


**Figure S2. Changes in the composition of the gut microbiota in response to STA supplementation.** (A) Changes in alpha diversity shown by Shannon index (A) and Simpson index (B). (C) Venn diagram of shared and non-shared OTUs. Relative abundance of *Akkermansia* (D) and *Alistipes* (E) in feces for the three groups. Comparison with the NCD group, * *P*<0.05, ** *P*<0.01, ns: no significance.


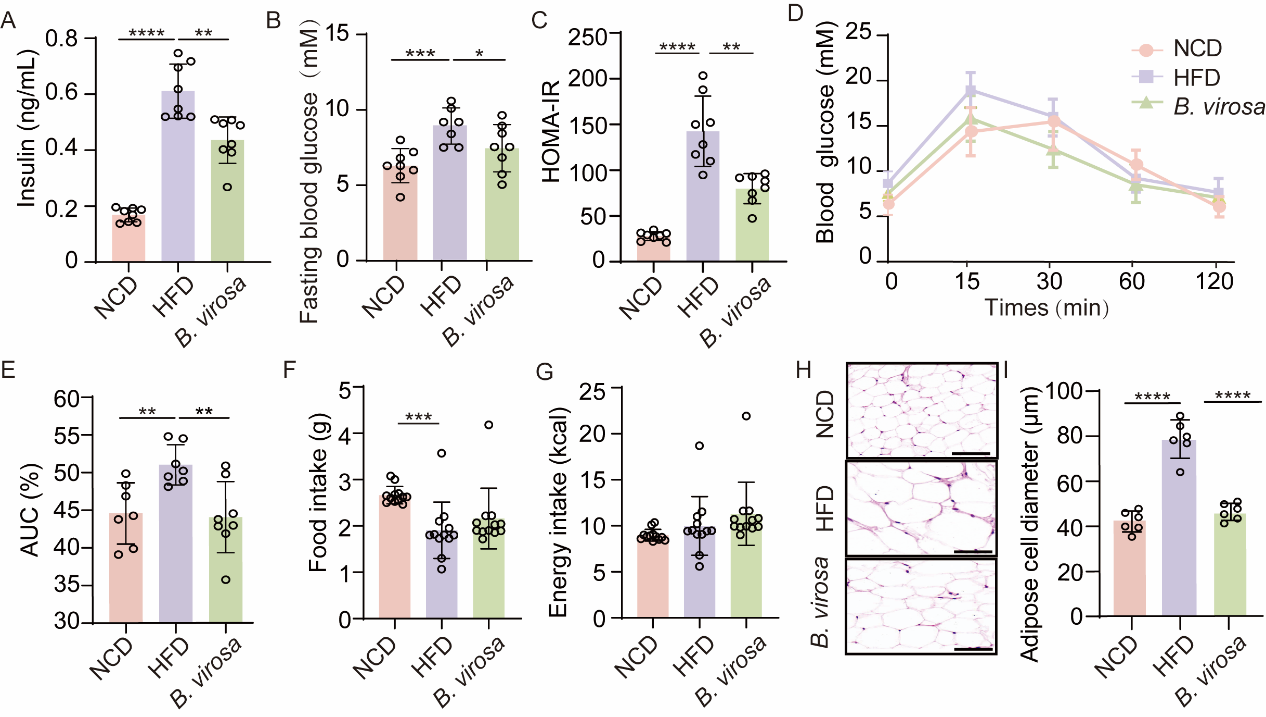


**Figure S3. Impact of *B. virosa* on body weight and glucose-lipid metabolism.** (A) Fasting insulin. (B) Fasting glucose. (C) HOMA-IR. (D) Plasma glucose profile measured during the IPGTT. (E) AUC for glucose tolerance test. (F) Food intake. (G) Energy intake. (H) Representative histological features of H&E-stained WAT tissues. Scale bar, 50 µm. (I) Adipocyte cell diameter. Comparison with the HFD group, * *P*<0.05, ** *P*<0.01, *** *P*<0.001, **** *P*<0.0001, ns: no significance.


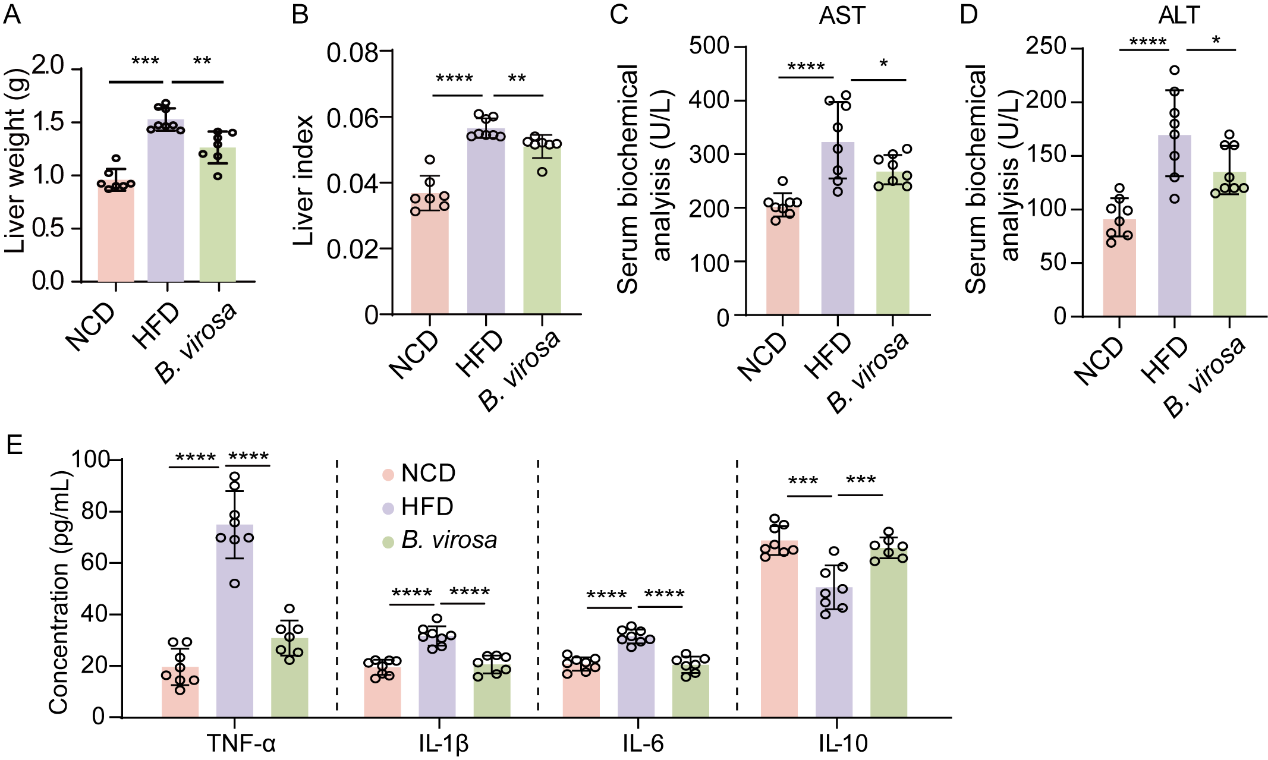


**Figure S4. Impact of *B. virosa* on MASLD and systemic inflammation.** (A) Liver weight. (B) Liver index. Biochemical analysis of serum levels of ALT (C) and AST (D). (E) Serum level of TNF-α, IL-1β, IL-6 and IL-10. Comparison with the HFD group, ** *P*<0.01, *** *P*<0.001, **** *P*<0.0001, ns: no significance.


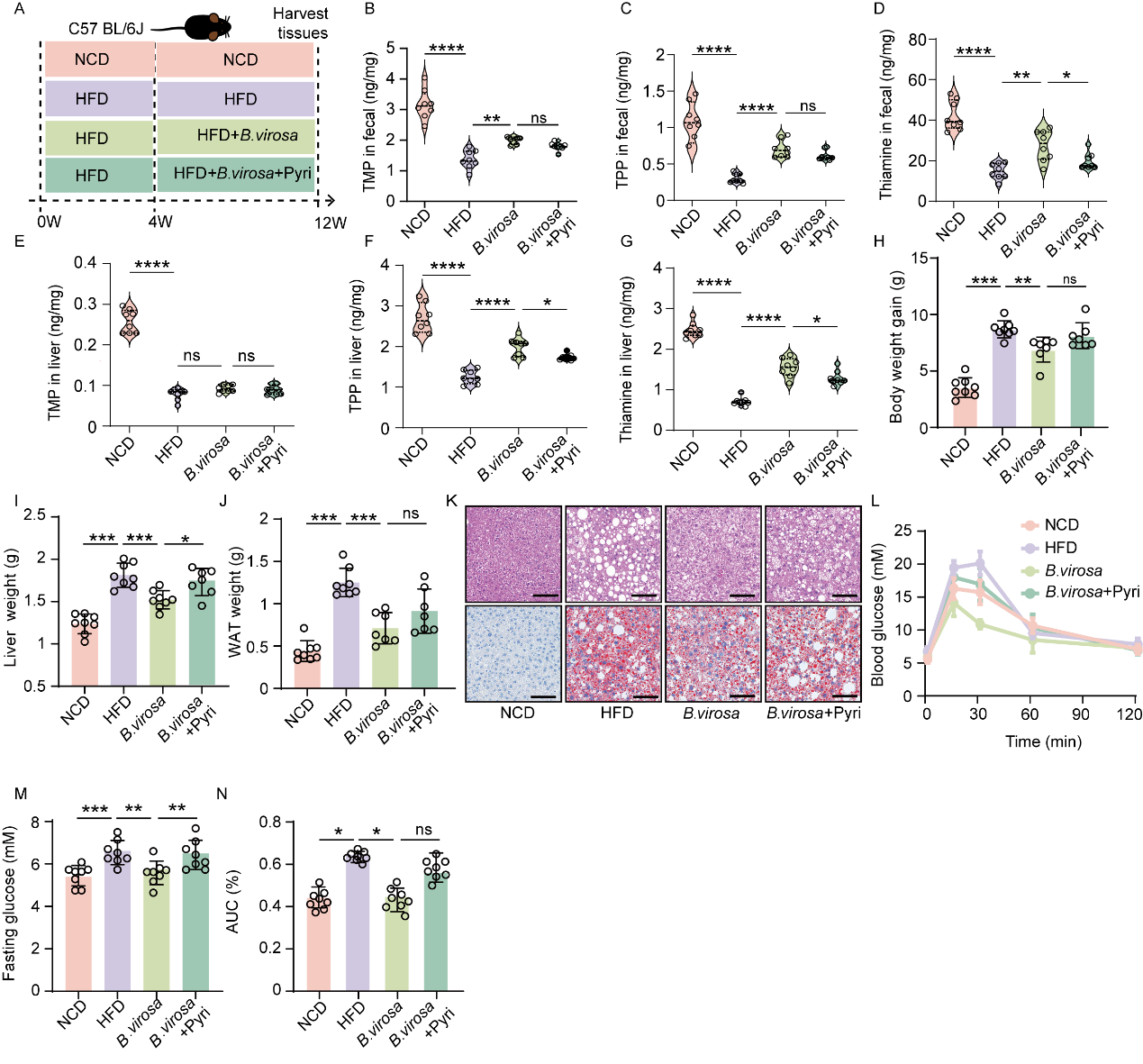


**Figure S5. Effect of *B. virosa* on hepatic thiamine levels under inhibitory conditions.** (A) Experimental design. n=8. The level of TMP (B), TPP (C) and thiamine (D) in fecal samples. Hepatic TMP (E), TPP (F) and thiamine (G) levels. (H) The final body weight gain. (I) Liver mass. (J) eWAT mass. (K) H&E staining and Oil Red O staining of liver tissues. (L) Plasma glucose profile measured during the IPGTT. (M) Fasting glucose. (N) AUC for glucose tolerance test. Comparison between indicated groups, * *P*<0.05, ** *P*<0.01, *** *P*<0.001, **** *P*<0.0001, ns: no significance.


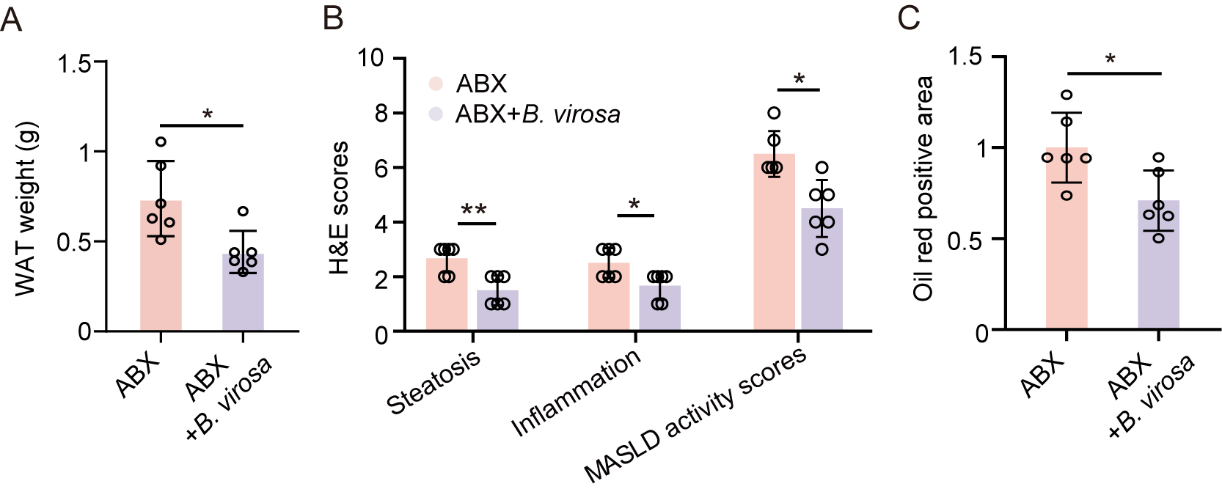


**Figure S6. The role of *B. virosa*-dependent effect on MASLD remission.** (A) eWAT mass. Quantitative analysis of hepatic H&E staining (B) and oil red O staining (C). Comparison with the ABX group, * *P*<0.05, ** *P*<0.01.


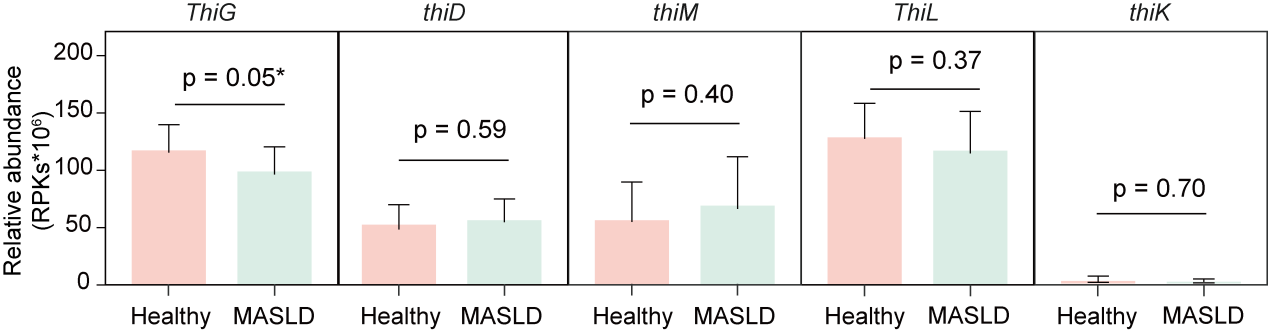


**Figure S7. Abundance of BCAAs metabolism-related genes in the human cohort.** Comparison with healthy individuals.

**Table S1. Diet details**

|  | NCD | HFD |
| --- | --- | --- |
| Carbohydrate (% of energy) | 64.00 | 20.00 |
| Protein (% of energy) | 20.00 | 20.00 |
| Fat (% of energy) | 7.00 | 60.00 |
| Energy (kcal/kg)  Ingredients, g/kg | 4000.00 | 5240.00 |
| Casein | 200.00 | 200.00 |
| Corn starch | 397.49 | 0.00 |
| Maltodextrin 10 | 0.00 | 125.00 |
| Sucrose | 100.00 | 68.8 |
| Dextrose | 132.00 | 0.00 |
| Cellulose | 50.00 | 50.00 |
| Soybean oil | 70.00 | 25.00 |
| Mineral mixture | 35.00 | 10.00 |
| Vitamin mixture | 10.00 | 10.00 |
| TBHQ | 0.01 | 0.00 |
| DL -methionine | 0.00 | 0.00 |
| L -cystine | 3.00 | 3.00 |
| Calcium carbonate | 0.00 | 5.50 |
| Cholesterol | 0.00 | 0.00 |
| Choline bitartrate | 2.50 | 2.00 |
| Chokeberry powder | 0.00 | 0.00 |
| Di-calcium phosphate | 0.00 | 13.00 |
| Calcium carbonate | 0.00 | 5.50 |
| Potassium citrate | 0.00 | 16.50 |
| Lard | 0.00 | 245.00 |

**Table S2. The Chemical and supply information for standard samples**

| **Name** | **CAS** | **Purity** | **Cat No.** | **Supplier** |
| --- | --- | --- | --- | --- |
| DL-leucine | 328-39-2 | 0.999 | C14629300 | Dr. Ehrenstorfer |
| DL-isoleucine | 443-79-8 | 0.995 | CDCT-C14429100 | Dr. Ehrenstorfer |
| DL-valine | 516-06-3 | 0.993 | C17899950 | Dr. Ehrenstorfer |
| Vitamin B1 | 154-87-0 | ＞99% | DRE-C17455000 | Dr. Ehrenstorfer |
| Vitamin B2 | 83-88-5 | ＞99% | CDCT-C16813600 | Dr. Ehrenstorfer |
| Vitamin B5 | 137-08-6 | ＞99% | CDCT-C15845000 | Dr. Ehrenstorfer |
| Vitamin B7 | 58-85-5 | ＞99% | DRE-C10625000 | Dr. Ehrenstorfer |

**Table S3. Graded elution program for targeted analysis vitamin B metabolites**

| Time/min | A (methanol)% | B (20mM ammonium formate )% |
| --- | --- | --- |
| 0.0 | 1 | 98 |
| 2.0 | 8 | 92 |
| 4.5 | 90 | 10 |
| 6.9 | 90 | 10 |
| 7.0 | 1 | 98 |
| 10.0 | 1 | 98 |

**Table S4. Graded elution program for targeted analysis of branched-chain amino acids**

| Time/min | A (100mM ammonium formate) % | B (95% acetonitrile water contains 0.3% formic acid) % |
| --- | --- | --- |
| 0.0 | 12 | 88 |
| 3.0 | 17 | 83 |
| 7.0 | 38 | 62 |
| 7.1 | 100 | 0 |
| 13.0 | 100 | 0 |
| 13.1 | 12 | 88 |
| 16.0 | 12 | 88 |

**Table S5. Primer sequences**

| Gene | Primer |
| --- | --- |
| *FASN-R* | 5’-GGCATCATTGGGCACTCCTT-3’ |
| *FASN-F* | 5’-GCTGCAAGCACAGCCTCTCT-3’ |
| *Acc1-R* | 5’-GATGAACCATCTCCGTTGGC-3’ |
| *Acc1-F* | 5’-GACCCAATTATGAATCGGGAGTG-3’ |
| *Srebp1c-R* | 5’-GGAGCCATGGATTGCACATT-3’ |
| *Srebp1c-F* | 5’-CCTGTCTCACCCCCAGCATA-3’ |
| *Scd1-R* | 5’-ATGCTCCAAGAGATCTCCAGTTCT-3’ |
| *Scd1-F* | 5’-ATGCTCCAAGAGATCTCCAGTTCT-3’ |
| *Acadl-R* | 5’-TACGGCACAAAAGAACAGATCG-3’ |
| *Acadl-F* | 5’-CAGGCTCTGTCATGGCTATGGT-3’ |
| *Cpt1A-R* | 5’-CTCAGTGGGAGCGACTCTTCA-3’ |
| *Cpt1A-F* | 5’-GGCCTCTGTGGTACACGACAA-3’ |
| *Acox1-R* | 5’-TAACTTCCTCACTCGAAGCCA-3’ |
| *Acox1-F* | 5’-AGTTCCATGACCCATCTCTGTC-3’ |
| *Fgfr1c-R* | 5’-GCCAGACAACTTGCCGTATG-3’ |
| *Fgfr1c-F* | 5’-ATTTCCTTGTCGGTGGTATTAACTC-3’ |
| *βklotho-R* | 5’-GATGAAGAATTTCCTAAACCAGGTT-3’ |
| *βklotho-F* | 5’-AACCAAACACGCGGATTTC-3’ |
| *Adiponectin-R* | 5’-AGACCTGGCCACTTTCTCCTCATT-3’ |
| *Adiponectin-F* | 5’-AGAGGAACAGGAGAGCTTGCAACA-3’ |
| *GAPDH-R* | 5’-AGGTCGGTGTGAACGGATTTG-3’ |
| *GAPDH-F* | 5’-TGTAGACCATGTAGTTGAGGTCA-3’ |
| *ThiG-F* | 5’-GCGTGAGAACCGCCAAAG-3’ |
| *ThiG-R* | 5’-AATAGGGTCGGGCAACAG-3’ |
| *ThiE-F* | 5’-TGGCGGCTAATGCTGATG-3’ |
| *ThiE-R* | 5’-ATGGCAACTAACGGATGG-3’ |
| *ThiL-F* | 5’-TGCCTGTGACCACTACGG-3’ |
| *ThiL-R* | 5’-TCTTCTTCATCGGCTTCC-3’ |
| *phoA-F* | 5’-TTTTACGGGCATCAACCT-3’ |
| *phoA-R* | 5’-TCAAACCACCTTCCACCA-3’ |
| *ALPI-F* | 5’-TTTTACGGGCATCAACCT-3’ |
| *ALPI-R* | 5’-TCAAACCACCTTCCACCA-3’ |

R: Reverse, F: Forward
